# Quantifying the Dynamics of Network Detachment Across Scientific, Technological, Commercial, and Pharmacological Domains

**DOI:** 10.1002/advs.78029

**Published:** 2026-10-04

**Authors:** Binglu Wang, Ching Jin, Chaoming Song, Johannes Bjelland, Brian Uzzi, Dashun Wang

**Affiliations:** ^1^ Center for Science of Science and Innovation Evanston Illinois USA; ^2^ Northwestern Institute on Complex Systems (NICO) Evanston Illinois USA; ^3^ Kellogg School of Management Evanston Illinois USA; ^4^ Centre for Interdisciplinary Methodologies University of Warwick Coventry UK; ^5^ Department of Physics University of Miami Coral Gables Florida USA; ^6^ Telenor Research and Development Fornebu Norway

**Keywords:** complex network, computational social science, computer science, innovation, network dynamics, phase transition, preferential detachment, science of science

## Abstract

Despite the vast literature on complex network dynamics, our understanding of the network detachment process remains limited. This understanding is essential for deeper insight into network evolution. Here, we analyze four large‐scale datasets that capture the structural patterns of detachment across scientific, technological, commercial, and pharmacological domains. Across these diverse domains, we uncover one simple pattern of preferential detachment in social systems, whereby the probability for individuals or organizations to disengage from a previously adopted practice correlates with the number of network neighbors who have already detached. To examine the structural consequences of this empirical pattern, we show that preferential detachment can fundamentally alter how networked systems disintegrate, inducing a phase transition in systems typically considered robust against detachment. We further derive an analytical framework to systematically understand the impact of preferential detachment on network dynamics, pinpointing the topological conditions under which it accelerates, decelerates, or leaves unchanged the breakdown of the network compared with random detachment. Together, these results demonstrate that the detachment dynamics follow simple yet reproducible patterns, deepening our quantitative understanding of detachment within networked social systems, with implications for the robustness and functioning of innovation communities.

## Graphical abstract

Across scientific, technological, commercial, and pharmacological domains, individuals and organizations disengage from a previously adopted practice not at random, but as their network neighbors detach. The probability of detachment rises with the number of neighbors who have already disengaged. Preferential detachment reshapes network disintegration, inducing a phase transition in systems typically considered robust against detachment.

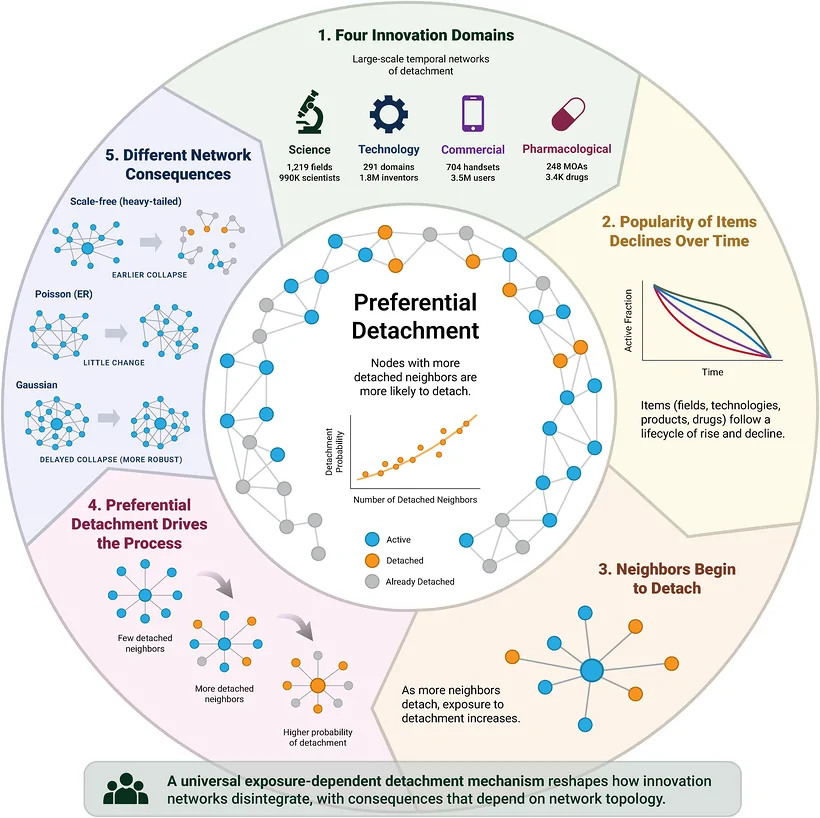

## 1 Introduction

Over the past several decades, research on complex networks has transformed our understanding of how interconnected systems form and expand across a diverse range of domains, from scientific collaboration and technological adoption to online information diffusion [1, 2, 3, 4, 5, 6, 7, 8, 9, 10, 11, 12, 13, 14]. A fundamental property of many networked systems is that popularity or activity eventually decays over time [15, 16, 17, 18, 19]. While the growth of networks has been extensively studied, the counterpart of this process—how networks disconnect through the disengagement or detachment of nodes—has received relatively little attention, especially within social contexts [16, 20], leaving open important questions about the dynamics of network detachment.

Existing research on network breakdown largely stems from studies of non‐social systems such as power grids, the Internet, financial infrastructures, and ecological food webs [2, 3, 21, 22]. In these domains, node removal is typically modeled as resulting from random failures, targeted attacks, or optimal percolation strategies, with a focus on structural robustness and systemic resilience [23, 24]. While these frameworks provide valuable insights, they abstract away from a defining feature of social systems: detachment is rarely random. Instead, real‐world social systems may exhibit critical network dynamics that arise from socially embedded decisions, shaped by peer behavior, structural interdependencies, and shifting incentives [25]. Such socially mediated detachment can propagate through networks, producing fragmentation or even systemic collapse, yet its structural regularities remain largely understudied.

One key reason for this gap in social systems is the historical lack of large‐scale datasets that systematically capture the detachment dynamics of networks [16]. Disengagement decisions are rarely recorded with the same precision as adoption or engagement events, making it difficult to systematically analyze patterns of detachment. To address this limitation, we compile and analyze four large‐scale datasets spanning the scientific, technological, commercial, and pharmacological domains.

The first dataset (*D*_1_) captures the detachment dynamics of scientific fields as scientists shift their focus away from certain areas of research. Here we use the Microsoft Academic Graph data [26] to measure the fraction of publishing scientists in each field over time, allowing us to delineate the life cycle of 1,219 scientific fields from 1940 to 2015 and quantify the dynamics of scientists leaving a field (Section S1.1). Our second dataset (*D*_2_) records the dynamics of inventors shifting across patenting domains, including 6.9 m patents granted by the United States Patent and Trademark Office from 1976 to 2015. Similar to *D*_1_, here we trace the life cycles of 291 patenting domains, approximated by Cooperative Patent Classification (CPC) codes, and measure the proportion of inventors in each domain over time (Section S1.2). For *D*_1_ and *D*_2_, we also construct the collaboration network using co‐authorship and co‐inventor information, respectively [4].

Our third dataset (*D*_3_) focuses on a commercial domain and traces the individual usage history of mobile handsets within a European country (704 types of handsets by 3.5 m users), allowing us to quantify the detachment dynamics of mobile handsets through changes in daily active users over a period of nine years (2006–2014). In addition, we collect anonymized phone call records among 1.7 m users within a three‐month period (October 1, 2013‐January 1, 2014) to approximate the social network among a subset of users for the network‐based analyses (Section S1.3). Lastly, we construct our fourth dataset (*D*_4_) tracing the dynamics of pharmacological technologies and analyze the detachment of drug mechanisms, i.e., biological mechanisms of action (MOAs), as pharmaceutical organizations shift their focus away from certain MOAs in drug development. Here we use the Cortellis Drug Discovery Intelligence database, which contains the largest and most comprehensive drug development records in the world, covering 648 702 drugs over the past 25 years [27, 28]. To quantify the detachment dynamics of pharmacological technologies, we examine MOAs associated with each drug and measure the research and development (R&D) focus of pharmaceutical companies on these MOAs. In total, we trace 248 MOAs by 5133 pharmaceutical companies and measure the fraction of companies using each MOA in drug development. We also calculate the similarity in market focus among pharmaceutical companies to approximate the underlying proximity between two companies (Section S1.4). The item‐specific relational networks exhibit heterogeneous, fat‐tailed degree distributions across all four empirical domains (Section S2.1). Together, these four datasets offer us an unprecedented opportunity to examine the temporal life‐cycle dynamics and the structural pattern in which the detachment processes unfold.

## 2 Results

Figure 1A,D,G,J plots the popularity dynamics of different scientific fields, patenting domains, mobile handsets, and drug MOAs, respectively. All four domains exhibit a similar rise‐and‐fall pattern, demonstrating that both the adoption and detachment processes are important for understanding these life‐cycle dynamics. Here we focus on the declining phase of each item and measure the rate of detachment ν as a function of time (*t*). We find that, across scientific fields, patenting domains, mobile handsets, and drug MOAs, the average detachment probability (ν) systematically increases with time (*t*) (Figure 1B,E,H,K). Going beyond the aggregate estimates, we test if the observed trend holds for individual scientific fields, patenting domains, mobile handsets, or drug MOAs. In particular, for each item, we build panel data containing all of its users, fit this panel data using linear regression, and estimate the slope of the fit for each item, to approximate how its detachment rate changes as a function of time (Figure 1C,F,I,L). We find that the majority of scientific fields (98%), patenting domains (92%), mobile handsets (91%), and drug MOAs (84%) can be approximated by a positive slope, suggesting the detachment probability of different items appears to trend upward in a rather ubiquitous manner.

**FIGURE 1:**
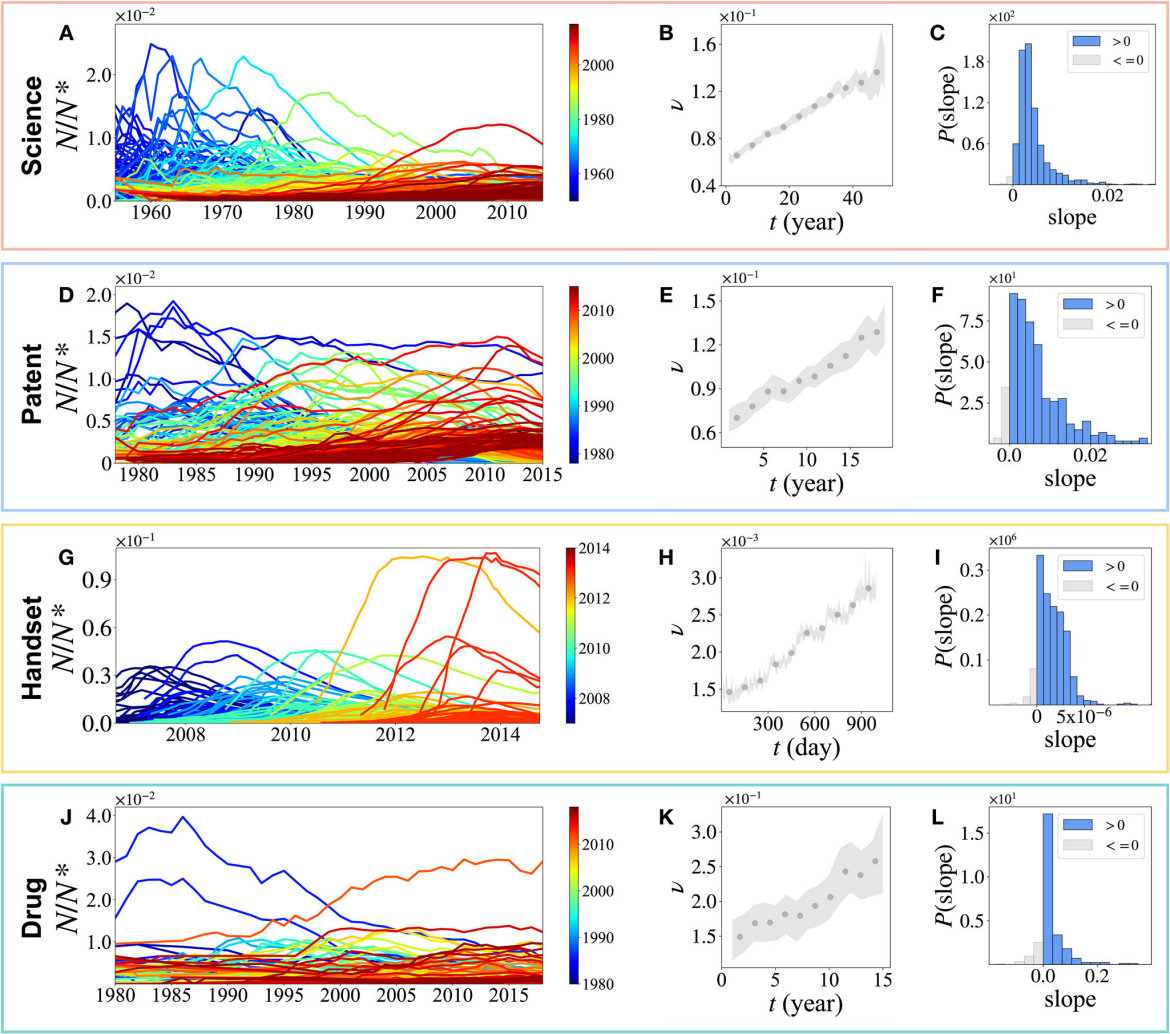
Temporal dynamics of innovation detachment. (A,D,G,J) Popularity dynamics ($\frac{N}{N^{*}}$) of scientific fields (A), patenting domains (D), mobile handsets (G), and drug MOAs (J). Here we calculate the proportion of scientists and inventors in each field, the ratio of active users for each mobile handset, and the fraction of pharmaceutical organizations focusing on each MOA. Each line represents a scientific field (A), patenting domain (D), handset type (G), or drug MOA (J). The color code corresponds to peak dates (when the population reaches its maximum), shifting from blue to red. (B,E,H,K) The average detachment probability $\nu \equiv \frac{\Delta N^{-}}{N}$ as a function of time *t* for all scientific fields (B), patenting domains (E), mobile handsets (H), and drug MOAs (K), showing that the detachment rate, on average, increases with time. The shaded region represents a 95% confidence interval. (C,F,I,L) We estimate the relationship between the detachment probability and time for individual scientific fields (C), patenting domains (F), mobile handsets (I), and drug MOAs (L), and we plot the distributions of the estimated slope coefficients.

To elucidate the relationship between detachment rates and underlying network structures, we further test the hypothesis that detachment processes follow Poissonian dynamics. This is a prevailing assumption in computational models, often derived from epidemiological frameworks [5, 29, 30, 31, 32, 33, 34, 35, 36, 37], and it has shown initial success in modeling substitutive systems [5]. This assumption reflects the idea that items follow a characteristic lifespan and that the probability of detachment is independent of the behavior of an individual's network neighbors. To test this implication, we control for potential temporal effects by restricting our measurements to a short time interval beginning around each item's half‐life (i.e., the stage at which its popularity has fallen to half of its peak), where detachment turnover is most active and a common life‐cycle stage is shared across items with different lifespans (Figure 2 and Section S2.2). We verify that our results are robust to this choice of time window (Section S2.3). For each item *i*, we consider its item‐specific relational network, namely the subnetwork of the underlying relational network formed by users associated with *i*, and divide users into two groups depending on whether detachment events have occurred among their network neighbors (*r_i_* > 0 vs. *r_i_* =  0) within the observation window. We find that the distributions of detachment rates for the two groups show visibly different patterns (Figure 2A–D), with the distribution for the first group (*r_i_* > 0) shifting systematically to the right of the *r_i_* =  0 group. These findings suggest that the detachment rate tends to be higher when detachment events have occurred among an individual's network neighbors. We then test whether the detachment probability (ν_*i*_) increases with the number of network neighbors who have also detached from the item (*r_i_*). Figure 2E–H shows four case studies, where we measure the rate of detachment as a function of *r_i_* for one selected example in each dataset. Averaged curves across all items in each domain show similar upward trends, confirming that the representative examples are consistent with population‐level patterns (Section S2.4). Across these four cases, ν_*i*_ shows a positive relationship with *r_i_*, indicating that the detachment probability appears to increase with the number of network neighbors who have detached. By contrast, in a null model of random detachment, ν_*i*_ is independent of *r_i_* (Figure 2E–H, grey dashed line). To systematically test across different items in different domains (i.e., scientific fields, patenting domains, mobile handsets, and drug MOAs), we measure ν_*i*_ and *r_i_* for each item in the four datasets, and we fit them with a linear ansatz, finding that a substantial fraction of scientific, technological, commercial, and pharmacological items exhibit a positive slope (73%, 70%, 79%, and 70%, respectively) (Figure 2I–L). This pattern also remains robust when exposure is normalized by degree, *r_i_*/*k_i_*, indicating that the result is not driven solely by the raw number of detached neighbors (Section S2.5).

**FIGURE 2:**
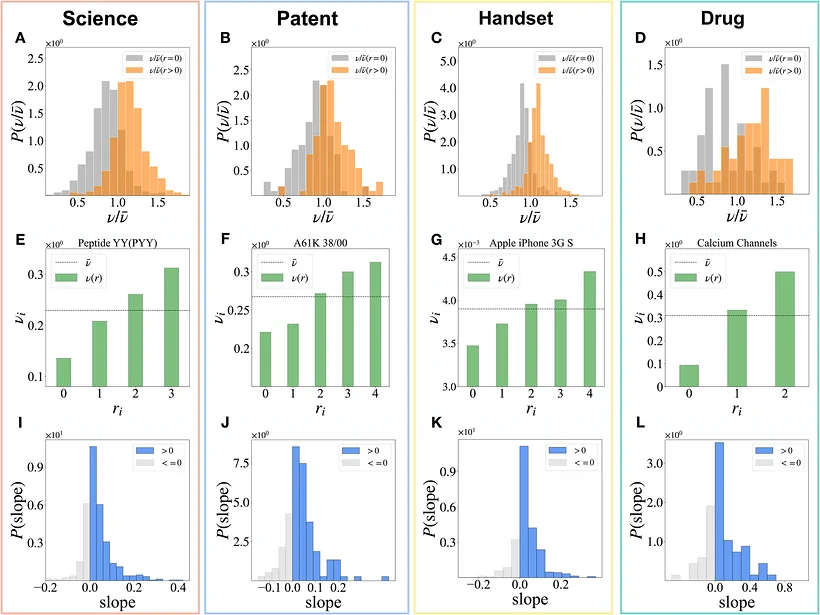
Detachment and the underlying social network. (A–D) Distributions of detachment probability plotted separately for people whose network neighbors have detached (orange) or not (grey). Here we control for temporal effects by taking a short time interval for our measurements (one year for the scientific fields, patenting domains, and drug MOAs, and 100 days for the mobile handsets). To maintain consistency of measurements across the four domains, we begin our measurements around the half‐life of each life cycle, i.e., when the item's popularity falls to half of its peak, which provides a comparable life‐cycle stage across items with different absolute lifespans; robustness checks using alternative reference points are reported in Section S2.3. We find the two distributions deviate from each other, suggesting that the underlying network further plays a role in detachment dynamics. (E–H) Detachment probability ν_*i*_ as a function of *r_i_* for one case selected from each domain as an illustrative example (*Peptide YY* for scientific fields, *A61K 38/00* for patenting domains, *iPhone 3GS* for handsets, and *Drugs Targeting Calcium Channels (Voltage‐Gated)* for drug MOAs). The four cases were selected as recognizable illustrative examples with sufficient observations across multiple values of *r_i_* for stable visualization. The dashed line indicates the null model, where detachment probability is independent of how many network neighbors have detached (*r_i_*). (I–L) We estimate the relationship between the detachment probability ν_*i*_ and *r_i_* for all scientific fields (I), patenting domains (J), mobile handsets (K), and drug MOAs (L), and we plot the distribution of the coefficients, finding that a vast majority of them can be approximated by a positive slope.

Overall, these empirical findings reveal a systematic pattern that we refer to as preferential detachment: the probability that an individual disengages increases with the number of detachments among their network neighbors. This observation contrasts with the Poissonian assumption that underlies most prevailing computational frameworks for modeling network detachment dynamics. We use the term “preferential” in a statistical sense, rather than to imply conscious preference or a specific causal mechanism. By analogy to preferential attachment, it denotes a non‐uniform detachment probability with respect to a structural quantity, here local exposure to prior detachment among network neighbors. We also distinguish this usage from prior uses of “preferential detachment” in network growth and rewiring models, where the term often refers to degree‐dependent edge removal, link rewiring, node deletion, or the loss of structural connections [38, 39, 40, 41]. Here detachment is defined as item‐specific disengagement conditioned on neighbors’ detachment states. Most importantly, the scientific, technological, commercial, and pharmacological domains we studied differ substantially in their scope, scale, temporal resolution, and nature. Yet despite these clear differences, and the myriad factors that might affect the decisions of individuals and organizations to detach from an innovative pursuit [42, 43, 44, 45, 46, 47], the patterns of preferential detachment show remarkable consistencies across these rather diverse domains.

The concept of preferential detachment challenges the Poissonian assumption that has traditionally underpinned computational models of network decline. At the same time, it connects to several important ideas in social science, from peer effects and network externalities [44, 48, 49, 50, 51, 52] to the theory of creative destruction [53] to vacancy chains [47, 54]. Interestingly, preferential detachment mirrors preferential attachment [1, 55, 56, 57, 58], the well‐established mechanism governing network growth that generates scale‐free structures with heterogeneous degree distributions. If preferential attachment governs how networks grow, preferential detachment may help explain how they decline. This parallel raises a natural question: what structural consequences might follow from this empirically observed detachment pattern?

To examine one such consequence, we study how preferential detachment reshapes network structure during periods of decline. We incorporate this empirical pattern into a minimal network model. Specifically, we approximate disengagement as node removal and formalize a detachment process on a network with *N* nodes. At each time step, node *i* is removed with probability:

(1) $$\pi_{i}=\frac{\alpha r_{i}+\left(1\;-\;\alpha \right)}{\sum_{j\in S}\;\alpha r_{j}+\left(1\;-\;\alpha \right)},$$

where *r_i_* denotes the number of neighbors of node *i* that have already been detached, α captures the strength of preferential detachment, and *S* denotes the set of surviving nodes. When α  =  0, nodes are removed randomly with the same probability, and Equation (1) recovers the existing modeling framework of random detachment. As α increases, node removal becomes increasingly conditioned on local detachment in the network. Estimating an empirical analogue of the preferential‐detachment strength further shows positive item‐level α values across all four domains (Section S2.6), providing a direct empirical bridge between the observed exposure–detachment relationship and the model parameter.

To analytically investigate the process, we adopt the heterogeneous mean‐field approximation (HMA) and derive a self‐consistent condition for the critical threshold (*f_c_*) under preferential detachment. The critical point satisfies:

(2) $$f_{c}=1-\frac{\left\langle k\right\rangle \sum_{k}P_{k}h{\left(f_{c}\right)}^{k}}{\sum_{k}P_{k}\left(k^{2}-k\right)h{\left(f_{c}\right)}^{k-2}}=1-\frac{G_{0}\left(h\left(f_{c}\right)\right)\left\langle k\right\rangle }{G_{0}^{\prime \prime }\left(h\left(f_{c}\right)\right)}$$

where $G_{0}\left(x\right)\equiv \;\sum_{k}P_{k}x^{k}$ and $G_{1}\left(x\right)\equiv \sum_{k}\frac{1}{\left\langle k\right\rangle }\left(k+1\right)P_{k+1}x^{k}$ are the standard generating functions of the degree distribution. The auxiliary function *h*(*f*), which captures the evolving local survival probability of edges during detachment, follows the differential equation:

(3) $$\begin{array}{c} \frac{dh}{df}=\frac{-\alpha h\;G_{0}^{2}\left(h\right)+\alpha \;G_{0}\left(h\right)G_{1}\left(h\right)\left(1-f\right)}{\begin{array}{c} \alpha \;\left(1-f\right)h\;G_{0}\left(h\right)G_{0}^{\prime }\left(h\right)-\alpha \;{\left(1-f\right)}^{2}G_{0}^{\prime }\left(h\right)G_{1}\left(h\right)\\ \;+\left(1-\alpha \right)\left(1-f\right)G_{0}^{2}\left(h\right) \end{array}}, \end{array}$$

with initial condition *h* (*f*_0_)= 1.

Together, Equations (2) and (3) provide a self‐consistent percolation threshold under preferential detachment. When α  =  0, preferential detachment reduces to random detachment and Equation (2) collapses to the canonical Molloy–Reed criterion. For scale‐free networks with γ < 3, the canonical criterion predicts no nontrivial phase‐transition point below complete removal when expressed as the removed‐node fraction, $f_{c}to1$. The situation changes qualitatively under preferential detachment (α > 0). As detachment progresses, *h*(*f*) drops below 1, which makes the factor $\sum_{k}P_{k}\left(k^{2}-k\right)h{\left(f_{c}\right)}^{k-2}$ converge and thereby yields a finite critical *f_c_* <  1 satisfying Equation (2). Thus, regardless of how weak the preference is, preferential detachment restores a genuine phase transition in scale‐free networks. In other words, even an infinitesimal amount of preferential detachment fundamentally changes the breakdown dynamics: a phase transition emerges that is absent in the random‐detachment case.

To verify this theoretical prediction, we first simulate the detachment process on a specific scale‐free network ($P_{k}\;∼\;k^{-\gamma }$, with γ  =  2.5), and we study its robustness to random detachment (α  =  0) and preferential detachment (α  =  0.5). Specifically, we trace the size of the giant component (*S*) as a function of the fraction of nodes removed (*f*). We find that under random detachment (Figure 3A, grey line), the network shows a high degree of robustness [2, 29, 37], as its giant component remains connected until all nodes are removed. If, however, the system undergoes preferential detachment (Figure 3A, red line), a fundamentally different pattern emerges. First, conditional on removing the same number of nodes, the network breaks down much more quickly than under random detachment. More importantly, the network appears to undergo a critical process and become fragmented when the removed node fraction exceeds a percolation threshold (*f_c_*).

**FIGURE 3:**
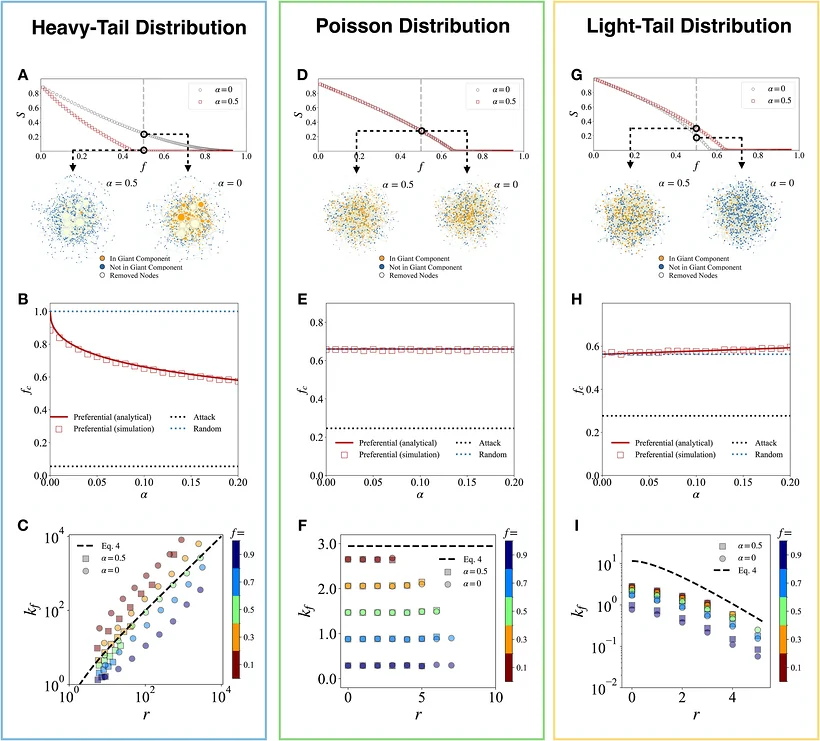
Preferential detachment across different network topologies. Simulations of detachment dynamics on networks with different degree distributions: a scale‐free network with a heavy‐tailed degree distribution (exponent γ  =  2.5; A–C), a network with a Poisson degree distribution (D–F), and a light‐tailed network with a normal degree distribution (G–I), all with the same average degree. Simulations are performed on networks of size *N*  =  10^6^ and averaged over 20 realizations. (A,D,G) Size of the giant component *S*, measured as the fraction of remaining nodes, as a function of the fraction of nodes removed (*f*), under preferential detachment (α  =  0.5), and under random detachment (α  =  0). Network visualizations show each network when half of its nodes have detached (*f*  =  0.5) and under preferential (α  =  0.5) and random (α  =  0) detachment. Here the nodes that have detached are shown in grey; among the remaining nodes, those in the giant connected component are shown in orange, and all others are shown in blue. (B,E,H) Critical fraction of node removal as a function of α for networks with heavy‐tailed (B), Poisson (E), and light‐tailed (H) degree distributions. For scale‐free networks (γ  =  2.5), simulated results under preferential detachment are shown as red squares, with the corresponding analytical prediction shown as a solid orange line. Results for targeted attacks on scale‐free networks (black dotted line) and random detachment (blue dotted line) are shown for comparison. We repeat the same analyses for networks with a Poisson distribution in (E) and a light‐tailed degree distribution in (H). (**C,F,I)** Relationship between the current degree *k_f_* and the number of detached neighbors *r* at different time snapshots (*f*  =  0.1,  0.3,  0.5,  0.7,  0.9), obtained from Monte Carlo simulation under random detachment (circles) and preferential detachment (α  =  0.5; squares) on heavy‐tailed (C), Poisson (F), and light‐tailed (I) networks. The dashed black line indicates the asymptotic behavior predicted by Equation (4) in the large‐*r* regime (see Section S4 for details).

We further simulate the preferential detachment process with different α (Figure 3B), and simulation results show excellent agreement with our analytical calculations derived from Equation (2). To further illuminate the process, we visualize the network when half of the nodes are removed (*f*  =  0.5) for both processes (subpanels in Figure 3A). While the two networks have the same number of nodes remaining, they show markedly different patterns. Whereas a giant component composed of high‐degree nodes persists under random detachment (α  =  0), the giant component in the preferential detachment case (α  =  0.5) only contains a small number of low‐degree nodes. This highlights a key difference between random and preferential detachment on scale‐free networks: compared with random detachment, hubs become disproportionately vulnerable under preferential detachment.

These results align closely with the network science literature [2, 5, 16, 29, 37, 55, 56, 57, 58, 59, 60, 61, 62, 63, 64, 65, 66, 67, 68, 69], which has extensively examined the importance of hubs in maintaining the robustness of networks. When hubs are deliberately targeted, the network collapses early (Figure 3B, dotted line). This raises a natural question: is preferential detachment equivalent to a targeted hub‐attack mechanism? To answer this question, we further examine preferential detachment on two additional network topologies: Erdős–Rényi (ER) networks with a Poisson degree distribution and a Gaussian network with a normal degree distribution. If preferential detachment is merely a hub attack, a similar earlier onset of phase transition should be expected for both networks (Figure 3E,H, black dotted line) [2, 56, 65].

However, Equation (2) predicts different behavior. Specifically, for ER networks, *G*_0_ (*x*) = *G*_1_ (*x*) = *e* ^〈*k*〉(*x* − 1)^ . Substituting this into Equation (2) yields the canonical Molloy–Reed criterion. In other words, preferential detachment follows indistinguishable dynamics from random detachment for the ER network. Interestingly, for Gaussian networks with degree distributions narrower than Poisson, preferential detachment induces an even later percolation transition than random detachment (Figure 3G), where the critical point is delayed as α increases (Figure 3H). Both predictions are further confirmed by simulation results (Figure 3D–I). These results indicate that preferential detachment is not a targeted hub‐attack mechanism. Instead, it has highly non‐trivial effects on different network topologies, highlighting a novel form of critical behavior and motivating a closer examination of the underlying mechanism.

To further understand how the percolation threshold shifts for different network topologies, we examine the relationship between a node's number of detached neighbors (*r*) and its current degree (*k_f_*) after a fraction *f* of nodes has detached. We first measure *k_f_* vs. *r* at different levels of *f* for the scale‐free network (Figure 3C), finding that the two quantities follow an asymptotically linear relationship, suggesting that nodes with larger *r* are also characterized by a higher remaining degree, mimicking the attack processes (see Section S4 for details). However, when we repeat the analyses on ER networks, we find that *k_f_* (*r*) remains constant for any given *r* (Figure 3F) under preferential detachment, as it does under random detachment. Because connections in ER networks are formed randomly, the number of a node's detached neighbors is statistically independent of its remaining degree during the detachment process. While nodes with higher *r* are more likely to be selected under preferential detachment, the corresponding *k_f_* is random for ER networks, explaining why preferential detachment on ER networks follows the same dynamics as random detachment.

Mathematically, the asymptotic behavior of *k_f_* (*r*) as r→∞ can be directly calculated by our model:

(4) $$k_{f}\left(r\right)∼\left(r+1\right)\frac{P_{k}\left(r+1\right)}{P_{k}\left(r\right)},$$

suggesting that this key relationship is governed by the original degree distribution of the network *P*(*k*) (see Section S4 for details). For the standard degree distribution considered here, whose tails are eventually decreasing in the large‐*r* regime, we have $\frac{P_{k}\left(r+1\right)}{P_{k}\left(r\right)}<1$. Equation (4) therefore predicts that *k_f_* (*r*) is asymptotically bounded by a linear scaling. For example, it saturates to the linear scaling bound for scale‐free networks. However, for the Poisson degree $P\left(k\right)∼\frac{\lambda^{k}}{k!}$, Equation (4) predicts $k_{f}∼\lambda$, indicating *k_f_* does not change with *r*. Thus, the Poisson degree distribution serves as a key division here. If the degree distribution has a heavier tail than the Poisson distribution, such as power‐law distributions, *k_f_* increases with *r*. Hence, in such networks, hubs are more likely to be removed under preferential detachment, leading to an earlier onset of the phase transition. By contrast, for networks with a narrower degree distribution than the Poisson distribution, such as normal distributions, there is a negative correlation between *k_f_* and *r* (Figure 3I), where once nodes with higher *r* are removed, preferential detachment actually targets nodes with lower current degrees, thus inducing an even slower percolation than random detachment. Table 1 summarizes the predictions of Equation (4) for several common networks.

**TABLE 1:** Summary of different network structures.

Network	Degree Distribution	$k_{f}\left(r\right){|}_{r\to \infty }$
scale‐free networks	*k* ^−γ^	*r*
ER networks	$\frac{\lambda^{k}e^{-\lambda }}{k!}$	λ
Gaussian networks	$e^{-\frac{1}{2}{\left(\frac{k-\mu }{\sigma }\right)}^{2}}$	0

## 3 Discussion

Taken together, this paper examines the declining dynamics across four distinct domains and makes three primary contributions. First, it empirically documents a simple yet ubiquitous phenomenon of preferential detachment governing the dynamics of network detachment. Across scientific, technological, commercial, and pharmacological domains, the probability of detachment is consistently associated with local exposure to prior detachment among network neighbors. These findings provide the central empirical contribution of the paper and suggest that detachment in social and innovation systems is not merely an individual‐level or item‐level life‐cycle process; it is also shaped by local network structure. Second, building on this empirical regularity, we incorporate preferential detachment into current modeling frameworks and find that its presence creates novel yet complex dynamics in the breakdown of the overall ecosystem, generating a new form of structural collapse in networked systems that are thought to be robust against random detachments. Third, we derive an analytical framework to systematically understand the impact of preferential detachment on network breakdown, pinpointing specific conditions under which it accelerates, decelerates, or leaves unchanged the breakdown of the network compared with random detachment, depending on the network topology. Overall, these results provide a new quantitative basis for understanding network detachment in social systems, which has implications for the robustness and functioning of interconnected communities.

While this paper synthesizes empirical evidence from four distinct domains to document the phenomenon of preferential detachment and its impact on the disintegration of networked communities, it is important to acknowledge a significant limitation: the study does not establish the origins of preferential detachment and its potential interplay with preferential attachment. For example, it is possible that the observed preferential detachment is an extension or byproduct of the preferential attachment mechanisms that govern the growth phase of the new communities. Although the findings of this paper remain valid irrespective of the specific origins of preferential detachment, further research into the connections between the growth and decline of networked communities could pave the way for an integrated framework for the whole life cycle of network dynamics, which would deepen our understanding of and ability to predict how networked communities evolve and dissolve.

Further, analyzing data across four diverse domains, this paper mainly focuses on universal detachment patterns that appear consistent across domains. At the same time, there are substantial heterogeneities both within and across domains. For example, scientists often have considerable autonomy in selecting research fields, whereas inventors typically operate under firm‐driven managerial strategies. While both scientists and inventors act as originators of ideas, mobile handset users function primarily as consumers. In contrast, the drug‐development dataset reflects organizational (firm‐level) decisions, where companies shift their R&D focus across different mechanisms of action.

The four domains also differ in the nature of their underlying relational networks. Scientific fields and patenting domains are based on collaboration networks, and mobile handsets are based on direct communication ties, whereas drug MOAs are based on similarity in market focus among pharmaceutical organizations. The persistence of preferential detachment across these different relational settings suggests a common exposure‐dependent pattern, while variation in the estimated strength of detachment across domains indicates that the degree of “stickiness” may depend on the type of actors, ties, and institutional context.

Observing consistent detachment patterns across these contextually distinct domains highlights important underlying commonalities in how detachment unfolds and further suggests fruitful directions for future research. For example, which factors tend to be associated with a higher or lower α parameter? Beyond the universal patterns presented in this paper, insights into the heterogeneity in the detachment processes may further our understanding of the potential mechanisms behind the detachment dynamics.

Our framework also connects to recent work on dynamical processes in complex networks, including studies of strategy evolution on dynamic networks and the evolution of prosocial behavior in multilayer populations [70, 71, 72]. These studies show how network architecture, temporal variation, and multilayer coupling can shape microscopic behavioral dynamics. Our work is complementary to this literature. Rather than examining how strategies, behaviors, or ties co‐evolve on a fixed, multilayer, or time‐varying network, we focus on item‐specific detachment: how actors disengage from a focal scientific field, patenting domain, handset model, or drug mechanism of action as local detachment accumulates around them. This distinction is also important when relating our framework to temporal rich‐club structure. In much of the temporal‐network literature, an edge represents a social, communication, or interaction tie, and a hub refers to an actor who is highly connected in the broader temporal network. In our setting, by contrast, the empirical analyses are conducted on item‐specific relational networks: subnetworks of the underlying relational network formed by actors associated with a focal item. Therefore, a hub in our analysis is not necessarily a socially central actor in the general network, but an actor with many neighbors within the item‐specific participation network. Likewise, detachment does not imply disappearance from the broader network; it means leaving a focal field, domain, product, or mechanism of action. Temporal rich‐club stability and preferential detachment therefore capture different levels of network dynamics. Future work could further examine this relationship by stratifying detachment dynamics by degree class, k‐core level, and rich‐club membership, and by extending the present framework to explicitly multilayer or time‐varying network settings.

Overall, this paper demonstrates that the dynamics of detachment across scientific, technological, commercial, and pharmacological domains follow simple yet reproducible patterns, commonly governed by preferential detachment. The phenomenon of preferential detachment fundamentally alters the breakdown of networked ecosystems, with implications for the robustness and functioning of the underlying systems. Curiously, preferential detachment resembles behavior observed across a wide range of settings, including organizational strategy [43, 46], culture [45], financial markets [44], and healthcare [47], suggesting that the concept of preferential detachment may have broad relevance that goes beyond these examples.

## Author Contributions

B.W., C.J., C.S., B.U., and D.W. designed the research; B.W. and C.J. collected data and conducted empirical and analytical analyses with help from D.W., C.S., J.B., and B.U.; all authors discussed and interpreted results; B.W., C.J., and D.W. wrote the manuscript; all authors edited the manuscript.

## Conflicts of Interest

The authors declare no conflicts of interest.

## Supporting information




**Supporting File**: advs78029‐sup‐0001‐SuppMat.pdf.

## Data Availability

The scientific publication data used in this work are from Microsoft Academic Graph. The raw data were publicly available at https://www.microsoft.com/en‐us/research/project/microsoft‐academic‐graph. The patent data used in this work are from PatentsView, which is publicly available at https://patentsview.org/download/data‐download‐tables. The mobile phone data are not publicly available because they contain commercially sensitive information, but they are available from the corresponding author upon reasonable request. This work also uses drug data sourced from Cortellis, and researchers who wish to access these raw data should contact Cortellis directly. Data and code necessary for reproducing the main results are available at https://github.com/BW‐KH/preferential‐detachment.

## References

[1] A.‐L. Barabási and R. Albert , “Emergence of Scaling in Random Networks,” Science 286, no. 5439 (1999): 509–512, 10.1126/science.286.5439.509. 10521342

[2] R. Albert , H. Jeong , and A.‐L. Barabási , “Error and Attack Tolerance of Complex Networks,” Nature 406, no. 6794 (2000): 378–382, 10.1038/35019019. 10935628

[3] S. V. Buldyrev , R. Parshani , G. Paul , H. E. Stanley , and S. Havlin , “Catastrophic Cascade of Failures in Interdependent Networks,” Nature 464, no. 7291 (2010): 1025–1028, 10.1038/nature08932. 20393559

[4] M. E. Newman , “The Structure of Scientific Collaboration Networks,” Proceedings of the National Academy of Sciences 98, no. 2 (2001): 404–409, 10.1073/pnas.98.2.404. PMC14598 11149952

[5] C. Jin , C. Song , J. Bjelland , G. Canright , and D. Wang , “Emergence of Scaling in Complex Substitutive Systems,” Nature Human Behaviour 3, no. 8 (2019): 837–846, 10.1038/s41562-019-0638-y. 31285621

[6] D. J. Watts , “A Simple Model of Global Cascades on Random Networks,” Proceedings of the National Academy of Sciences 99, no. 9 (2002): 5766–5771, 10.1073/pnas.082090499. PMC122850 16578874

[7] J. A. Evans and J. G. Foster , “Metaknowledge,” Science 331, no. 6018 (2011): 721–725. 10.1126/science.1201765 21311014

[8] S. Fortunato , C. T. Bergstrom , K. Börner , et al., “Science of Science,” Science 359, no. 6379 (2018): aao0185, 10.1126/science.aao0185. PMC5949209 29496846

[9] D. Wang and A.‐L. Barabási , The Science of Science (Cambridge University Press, 2021), 10.1017/9781108610834.

[10] V. Colizza , A. Barrat , M. Barthélemy , and A. Vespignani , “The Role of the Airline Transportation Network in the Prediction and Predictability of Global Epidemics,” Proceedings of the National Academy of Sciences 103, no. 7 (2006): 2015–2020, 10.1073/pnas.0510525103. PMC1413717 16461461

[11] D. Brockmann and D. Helbing , “The Hidden Geometry of Complex, Network‐driven Contagion Phenomena,” Science 342, no. 6164 (2013): 1337–1342, 10.1126/science.1245200. 24337289

[12] J.‐P. Onnela and F. Reed‐Tsochas , “Spontaneous Emergence of Social Influence in Online Systems,” Proceedings of the National Academy of Sciences 107, no. 43 (2010): 18375–18380, 10.1073/pnas.0914572107. PMC2972979 20937864

[13] B. Wang , Z. Ma , D. Wang , B. Uzzi , et al., “Peer Review and the Diffusion of Ideas,” *arXiv* (2025): 2507.11825.

[14] F. Meng , J. Xie , J. Sun , et al., “Spreading Dynamics of Information on Online Social Networks,” Proceedings of the National Academy of Sciences 122, no. 4 (2025): 2410227122. 10.1073/pnas.2410227122 PMC11789049 39847317

[15] T. Kuhn , The Structure of Scientific Revolutions (University of Chicago Press, 1962).

[16] S. Saavedra , F. Reed‐Tsochas , and B. Uzzi , “Asymmetric Disassembly and Robustness in Declining Networks,” Proceedings of the National Academy of Sciences 105, no. 43 (2008): 16466–16471, 10.1073/pnas.0804740105. PMC2575443 18936489

[17] D. Wang , C. Song , and A.‐L. Barabási , “Quantifying Long‐term Scientific Impact,” Science 342, no. 6154 (2013): 127–132, 10.1126/science.1237825. 24092745

[18] Y. Yin and D. Wang , “The Time Dimension of Science: Connecting the Past to the Future,” Journal of Informetrics 11, no. 2 (2017): 608–621, 10.1016/j.joi.2017.04.002.

[19] C. Candia , C. Jara‐Figueroa , C. Rodriguez‐Sickert , A.‐L. Barabási , and C. A. Hidalgo , “The Universal Decay of Collective Memory and Attention,” Nature Human Behaviour 3, no. 1 (2019): 82–91, 10.1038/s41562-018-0474-5. 30932052

[20] E. D. Lee , C. P. Laubichler , M. D. Hamilton , et al., “Synthesis of Innovation and Obsolescence,” *arXiv* (2025): 2505.05182.

[21] A. G. Haldane and R. M. May , “Systemic Risk in Banking Ecosystems,” Nature 469, no. 7330 (2011): 351–355, 10.1038/nature09659. 21248842

[22] J. A. Dunne , R. J. Williams , and N. D. Martinez , “Network Structure and Biodiversity Loss in Food Webs: Robustness Increases with Connectance,” Ecology Letters 5, no. 4 (2002): 558–567.

[23] J. Gao , B. Barzel , and A.‐L. Barabási , “Universal Resilience Patterns in Complex Networks,” Nature 530, no. 7590 (2016): 307–312, 10.1038/nature16948. 26887493

[24] O. Artime , M. Grassia , M. De Domenico , et al., “Robustness and Resilience of Complex Networks,” Nature Reviews Physics 6, no. 2 (2024): 114–131, 10.1038/s42254-023-00676-y.

[25] R. Hill , Y. Yin , C. Stein , X. Wang , D. Wang , and B. F. Jones , “The Pivot Penalty in Research,” Nature 642 (2025): 999–1006. 10.1038/s41586-025-09048-1 PMC12222024 40437087

[26] A. Sinha , Z. Shen , and Y. Song , “An Overview of Microsoft Academic Service (MAS) and Applications,” in Proceedings of the 24th International Conference on World Wide Web (Association for Computing Machinery, 2015), 10.1145/2740908.2742839.

[27] B. Lou and L. Wu , “AI on Drugs: Can Artificial Intelligence Accelerate Drug Development? Evidence from a Large‐scale Examination of Bio‐pharma Firms,” MIS Quarterly 45, no. 3 (2021): 1451–1482, 10.25300/MISQ/2021/16565.

[28] J. L. Krieger , “Trials and Terminations: Learning from Competitors′ R&D Failures,” Management Science 67, no. 9 (2021): 5525–5548, 10.1287/mnsc.2020.3775.

[29] R. Pastor‐Satorras , C. Castellano , P. Van Mieghem , and A. Vespignani , “Epidemic Processes in Complex Networks,” Reviews of Modern Physics 87, no. 3 (2015): 925–979, 10.1103/RevModPhys.87.925.

[30] H. W. Hethcote , “The Mathematics of Infectious Diseases,” SIAM Review 42, no. 4 (2000): 599–653, 10.1137/S0036144500371907.

[31] S. V. Scarpino and G. Petri , “On the Predictability of Infectious Disease Outbreaks,” Nature Communications 10, no. 1 (2019): 898, 10.1038/s41467-019-08616-0. PMC6385200 30796206

[32] Z. Ruan , G. Iñiguez , M. Karsai , and J. Kertész , “Kinetics of Social Contagion,” Physical Review Letters 115, no. 21 (2015): 218702, 10.1103/PhysRevLett.115.218702. 26636878

[33] I. Iacopini , G. Petri , A. Barrat , and V. Latora , “Simplicial Models of Social Contagion,” Nature Communications 10, no. 1 (2019): 2485, 10.1038/s41467-019-10431-6. PMC6554271 31171784

[34] A. L. Hill , D. G. Rand , M. A. Nowak , and N. A. Christakis , “Infectious Disease Modeling of Social Contagion in Networks,” PLOS Computational Biology 6, no. 11 (2010): 1000968, 10.1371/journal.pcbi.1000968. PMC2973808 21079667

[35] G. F. de Arruda , G. Petri , and Y. Moreno , “Social Contagion Models on Hypergraphs,” Physical Review Research 2, no. 2 (2020): 023032.

[36] M. Karsai , G. Iñiguez , K. Kaski , and J. Kertész , “Complex Contagion Process in Spreading of Online Innovation,” Journal of The Royal Society Interface 11, no. 101 (2014): 20140694, 10.1098/rsif.2014.0694. PMC4223898 25339685

[37] R. Pastor‐Satorras and A. Vespignani , “Epidemic Spreading in Scale‐Free Networks,” Physical Review Letters 86, no. 14 (2001): 3200–3203, 10.1103/PhysRevLett.86.3200. 11290142

[38] S. Johnson , J. J. Torres , and J. Marro , “Nonlinear Preferential Rewiring in Fixed‐Size Networks as a Diffusion Process,” Physical Review E—Statistical, Nonlinear, and Soft Matter Physics 79, no. 5 (2009): 050104. 10.1103/PhysRevE.79.050104 19518399

[39] H. Brot , M. Honig , L. Muchnik , J. Goldenberg , Y. Louzoun , et al., “Edge Removal Balances Preferential Attachment and Triad Closing,” Physical Review E—Statistical, Nonlinear, and Soft Matter Physics 88, no. 4 (2013): 042815. 10.1103/PhysRevE.88.042815 24229233

[40] S. Laird and H. J. Jensen , “A Steady state Network Model with a 1/k Scale‐free Degree Distribution,” arXiv (2006): cond‐mat/0603199.

[41] S. Lim , C. E. Han , P. J. Uhlhaas , and M. Kaiser , “Preferential Detachment during human Brain Development: Age‐and Sex‐Specific Structural Connectivity in Diffusion Tensor Imaging (DTI) Data,” Cerebral Cortex 25, no. 6 (2015): 1477–1489, 10.1093/cercor/bht333. PMC4428296 24343892

[42] L. R. Burns and D. R. Wholey , “Adoption and Abandonment of Matrix Management Programs: Effects of Organizational Characteristics and Interorganizational Networks,” Academy of Management Journal 36, no. 1 (1993): 106–138, 10.2307/256514. 10123742

[43] H. R. Greve , “Jumping Ship: the Diffusion of Strategy Abandonment,” Administrative Science Quarterly 40, no. 3 (1995): 444–473, 10.2307/2393793.

[44] H. Rao , H. R. Greve , and G. F. Davis , “Fool's Gold: Social Proof in the Initiation and Abandonment of Coverage by Wall Street Analysts,” Administrative Science Quarterly 46, no. 3 (2001): 502–526, 10.2307/3094873.

[45] J. Berger and G. L. Mens , “How Adoption Speed Affects the Abandonment of Cultural Tastes,” Proceedings of the National Academy of Sciences 106, no. 20 (2009): 8146–8150, 10.1073/pnas.0812647106. PMC2688844 19416813

[46] V. Gaba and G. Dokko , “Learning to Let Go: Social Influence, Learning, and the Abandonment of Corporate Venture Capital Practices,” Strategic Management Journal 37, no. 8 (2016): 1558–1577, 10.1002/smj.2404.

[47] B. N. Greenwood , R. Agarwal , R. Agarwal , and A. Gopal , “The When and Why of Abandonment: The Role of Organizational Differences in Medical Technology Life Cycles,” Management Science 63, no. 9 (2017): 2948–2966, 10.1287/mnsc.2016.2490.

[48] N. A. Christakis and J. H. Fowler , “The Spread of Obesity in a Large Social Network over 32 Years,” New England Journal of Medicine 357, no. 4 (2007): 370–379, 10.1056/NEJMsa066082. 17652652

[49] M. L. Katz and C. Shapiro , “Network Externalities, Competition, and Compatibility,” The American Economic Review 75, no. 3 (1985): 424–440.

[50] S. Aral , L. Muchnik , and A. Sundararajan , “Distinguishing Influence‐based Contagion from Homophily‐driven Diffusion in Dynamic Networks,” Proceedings of the National Academy of Sciences 106, no. 51 (2009): 21544–21549, 10.1073/pnas.0908800106. PMC2799846 20007780

[51] S. Aral and D. Walker , “Identifying Influential and Susceptible Members of Social Networks,” Science 337, no. 6092 (2012): 337–341, 10.1126/science.1215842. 22722253

[52] M. A. Schilling , “Technology Success and Failure in Winner‐take‐all Markets: The Impact of Learning Orientation, Timing, and Network Externalities,” Academy of Management Journal 45, no. 2 (2002): 387–398, 10.2307/3069353.

[53] J. A. Schumpeter , Capitalism, Socialism, and Democracy (Harper & Brothers, 1942).

[54] H. C. White , Chains of Opportunity (Harvard University Press, 2013).

[55] R. Cohen , K. Erez , D. ben‐Avraham , and S. Havlin , “Resilience of the Internet to Random Breakdowns,” Physical Review Letters 85, no. 21 (2000): 4626–4628, 10.1103/PhysRevLett.85.4626. 11082612

[56] R. Cohen , K. Erez , D. ben‐Avraham , and S. Havlin , “Breakdown of the Internet under Intentional Attack,” Physical Review Letters 86, no. 16 (2001): 3682–3685, 10.1103/PhysRevLett.86.3682. 11328053

[57] A.‐L. Barabási , Network science (Cambridge University Press, 2016).

[58] M. Newman , Networks (Oxford University Press, 2018).

[59] D. S. Callaway , M. E. J. Newman , S. H. Strogatz , and D. J. Watts , “Network Robustness and Fragility: Percolation on Random Graphs,” Physical Review Letters 85, no. 25 (2000): 5468–5471, 10.1103/PhysRevLett.85.5468. 11136023

[60] M. Li , R.‐R. Liu , L. Lü , M.‐B. Hu , S. Xu , and Y.‐C. Zhang , “Percolation on Complex Networks: Theory and Application,” Physics Reports 907 (2021): 1–68, 10.1016/j.physrep.2020.12.003.

[61] A. Allard , B. M. Althouse , S. V. Scarpino , and L. Hébert‐Dufresne , “Asymmetric Percolation Drives a Double Transition in Sexual Contact Networks,” Proceedings of the National Academy of Sciences 114, no. 34 (2017): 8969–8973, 10.1073/pnas.1703073114. PMC5576788 28790185

[62] P. S. Dodds and D. J. Watts , “Universal Behavior in a Generalized Model of Contagion,” Physical Review Letters 92, no. 21 (2004): 218701, 10.1103/PhysRevLett.92.218701. 15245323

[63] D. Garcia , P. Mavrodiev , and F. Schweitzer , “Social Resilience in Online Communities: The Autopsy of Friendster,” in Proceedings of the first ACM Conference on Online Social Networks (Association for Computing Machinery, 2013), 10.1145/2512938.2512946.

[64] J. Török and J. Kertész , “Cascading Collapse of Online Social Networks,” Scientific Reports 7, no. 1 (2017): 16743. 10.1038/s41598-017-17135-1 PMC5711899 29196654

[65] A. Yehezkel and R. Cohen , “Degree‐Based Attacks and Defense Strategies in Complex Networks,” Physical Review E 86, no. 6 (2012): 066114, 10.1103/PhysRevE.86.066114. 23368011

[66] L. Tian , A. Bashan , D.‐N. Shi , and Y.‐Y. Liu , “Articulation Points in Complex Networks,” Nature Communications 8, no. 1 (2017): 1–9, 10.1038/ncomms14223. PMC5290321 28139697

[67] A. E. Motter and Y.‐C. Lai , “Cascade‐Based Attacks on Complex Networks,” Physical Review E 66, no. 6 (2002): 065102, 10.1103/PhysRevE.66.065102. 12513335

[68] K.‐I. Goh , D.‐S. Lee , B. Kahng , and D. Kim , “Sandpile on Scale‐Free Networks,” Physical Review Letters 91, no. 14 (2003): 148701, 10.1103/PhysRevLett.91.148701. 14611564

[69] I. Dobson , B. A. Carreras , V. E. Lynch , and D. E. Newman , “Complex Systems Analysis of Series of Blackouts: Cascading Failure, Critical Points, and Self‐Organization,” Chaos: An Interdisciplinary Journal of Nonlinear Science 17, no. 2 (2007): 026103, 10.1063/1.2737822. 17614690

[70] Q. Su , A. McAvoy , Y. Mori , and J. B. Plotkin , “Evolution of Prosocial Behaviours in Multilayer Populations,” Nature Human Behaviour 6, no. 3 (2022): 338–348, 10.1038/s41562-021-01241-2. 34980900

[71] Q. Su , A. McAvoy , and J. B. Plotkin , “Strategy Evolution on Dynamic Networks,” Nature Computational Science 3, no. 9 (2023): 763–776, 10.1038/s43588-023-00509-z. 38177777

[72] H. Chiba‐Okabe and J. B. Plotkin , “Social Learning with Complex Contagion,” Proceedings of the National Academy of Sciences 121, no. 49 (2024): 2414291121, 10.1073/pnas.2414291121. PMC11626147 39602255

