# Supplementary material for: Quantifying the Dynamics of Network Detachment Across Scientific, Technological, Commercial, and Pharmacological Domains
Source: Adv Sci (Weinh). 2026 Oct 4:e78029. Online ahead of print. doi: 10.1002/advs.78029 (PMC13634477; doi:10.1002/advs.78029)
Supplement: Supplementary file 1 [file ADVS-9999-e78029-s001.pdf]

Supplementary Information for Quantifying the Dynamics of Network Detachment Across Scientific, Technological, Commercial, and Pharmacological Domains

Binglu Wang[1,2,3*], Ching Jin[1,2,3,4*], Chaoming Song[5], Johannes Bjelland[6], Brian Uzzi[1,2,3] & Dashun Wang[1,2,3†]

[1] *Center for Science of Science and Innovation, 1800 Sherman Avenue, Evanston, IL 60201, USA*

[2] *Northwestern Institute on Complex Systems (NICO), 600 Foster Street, Evanston, IL 60208, USA*

[3] *Kellogg School of Management, 2211 Campus Drive, Evanston, IL 60208, USA*

[4] *Centre for Interdisciplinary Methodologies, University of Warwick, Coventry, CV4 7AL, United Kingdom*

[5] *Department of Physics, University of Miami, 1320 S Dixie Hwy, Coral Gables, FL 33146, USA*

[6] *Telenor Research and Development, Snarøyveien 30 N-1360 Fornebu, Norway*

**These authors contributed equally to this work.*

[†] *Correspondence should be addressed to D.W. (dashun.wang@kellogg.northwestern.edu)*

Contents

S1 Dataset Descriptions **4**

S1.1 D_1: Detachment in Scientific Fields 4

S1.2 D_2: Detachment in Patenting 5

S1.3 D_3: Detachment from Mobile Handsets 6

S1.4 D_4: Detachment in Pharmacological Innovation 7

S2 Empirical Methods **8**

S2.1 Network Structure of Different Social Systems 8

S2.2 Selecting Time Intervals to Measure the Structural Effect 11

S2.3 Robustness Check Across Different Time Intervals 11

S2.4 Robustness Check on Averaged Detachment Curves 13

S2.5 Robustness Check on Normalized Exposure, r_i/k_i 14

S2.6 Robustness Check on α Distribution 14

S3 Heterogeneous Mean-Field Theory **16**

S3.1 Method Description 16

S3.2 Express i_k as a Function of t 17

S3.3 Relationship Between i'_k and i_k 18

S3.4 Express i_k as a Function of f 19

S3.5 Molloy–Reed Criterion Method 20

S4 Relationship Between k_f and r **21**

S4.1 Relationship Between k_f and r in Different Network Structures 21

S4.2 k_f as a Function of r 25

1. Random Detachment ($\alpha = 0$) 25

2. Preferential Detachment ($\alpha > 0$) 30

S5 Supplementary References **40**

S1 Dataset Descriptions

S1.1 D_1: Detachment in Scientific Fields

The first dataset (D_1) tracks the detachment dynamics of scientific fields through the publication records of individual scientists. The data are drawn from the Microsoft Academic Graph (MAG), which contains approximately 172 million publications and their associated metadata[1]. We use the MAG field classification system to identify research domains[2].

Identifying detachment from scientific fields for each scientist: MAG organizes research fields in a hierarchical taxonomy. We focus on leaf-level fields (i.e., fields without child fields). When a field contains fewer than 500 publications or has more than three hierarchical parent levels, we instead use the corresponding parent fields to ensure sufficient observations and stable classifications. To determine when a scientist detaches from a field, we identify the year in which the scientist's primary research focus shifts away from that field after having worked in it for at least two consecutive years (Fig. S1). Specifically, we implement the following procedure: (1) compile all publications associated with each scientist that are linked to at least one leaf field; (2) for each year, identify the field in which the scientist published the largest number of papers and define it as the scientist's primary research focus for that year; and (3) construct the scientist's career trajectory based on the sequence of their annual primary research focuses. A detachment event occurs when a field no longer appears as the scientist's primary research focus in subsequent years. Our analysis includes scientists whose publishing careers span at least 10 years and who

have published at least 10 papers.

Overall, D_1 includes 990K scientists working across 1,219 scientific fields that emerged after 1940 and were studied by at least 500 scientists from 1940 to 2015.

S1.2 D_2: Detachment in Patenting

The second dataset (D_2) captures detachment dynamics in technological domains using 6.9M patents granted by the United States Patent and Trademark Office (USPTO) from 1976 to 2015. Technological domains are approximated using the Cooperative Patent Classification (CPC) system, focusing on CPC subgroup codes.

Identifying detachment from patenting domains for each inventor: Following a procedure similar to that used for D_1, we reconstruct each inventor's annual patenting focus. For each year, we identify the CPC subgroup in which the inventor produces the largest number of patents and define this subgroup as the inventor's primary patenting domain for that year. Based on these annual domain assignments, a detachment event occurs when an inventor ceases to focus their patenting activity on a previously dominant domain.

To ensure adequate coverage, we restrict the analysis to patenting domains involving at least 200 inventors during the observation period. The resulting dataset includes 123K inventors with patenting careers spanning more than five years across 291 technological domains from 1976 to

2015.

S1.3 D_3: Detachment from Mobile Handsets

The third dataset (D_3) captures the commercial domain of mobile handsets, recording individual usage history provided by a European telecommunication company. We identify detachment from mobile handsets by tracking changes in their daily active users from 2006 to 2014.

Identifying detachment from mobile handsets for each consumer: We determine the detachment from mobile handsets by each user through SIM card records, which provide clear timelines for when users change mobile phones. Specifically, we use the last usage day of a mobile handset as the user's detachment time from that handset.

We focus on mobile handsets used by more than 1,000 users. In total, D_3 records 3.5M users using 704 types of mobile handsets from 2006 to 2014.

For the network-based analyses, we approximate the communication network among mobile-phone users using anonymized call-detail records over a three-month window from October 1, 2013, to January 1, 2014. Two users are connected by an edge if they exchanged at least one phone call during this window. The network-based detachment analyses are conducted on the subset of 1.7M users observed in these call records, for whom both handset usage and communication ties can be measured. Users without communication-network observations are

retained in the aggregate handset life-cycle analyses but are excluded from analyses requiring network-neighbor information.

S1.4 D_4: Detachment in Pharmacological Innovation

The fourth dataset (D_4) examines detachment dynamics in pharmaceutical innovation using the Cortellis Drug Discovery Intelligence database. This database contains detailed information on global drug development pipelines and covers 648,702 drug candidates over the past 25 years.

Identifying detachment from mechanisms of action (MOAs) for each pharmaceutical organization: We characterize pharmacological innovation using biological mechanisms of action (MOAs) associated with drug candidates. For each pharmaceutical company, we track its portfolio of MOAs used in clinical development. A detachment event occurs when a pharmaceutical organization ceases to pursue a previously adopted MOA in clinical trials.

We focus on second-level or higher MOA categories adopted by at least 10 pharmaceutical organizations. The resulting dataset tracks 248 MOAs used by 5,133 pharmaceutical companies and measures the fraction of organizations pursuing each MOA in drug development.

S2 Empirical Methods

S2.1 Network Structure of Different Social Systems

The present study investigates the decline dynamics of robust network systems, which represent a predominant class of networks in social contexts[3]. As a first step, we examine the structural properties of the four empirical systems analyzed in this study (i.e., the scientific field dataset, patenting dataset, mobile handset dataset, and drug development dataset). Specifically, we plot the degree distributions of the corresponding social networks for each dataset (Fig. S2).

Across all four systems, we observe that the degree distributions exhibit heavy-tailed patterns, consistent with the heterogeneous connectivity commonly observed in real-world social networks. These findings confirm that the empirical systems examined in this study belong to the class of heterogeneous networks that have been widely studied in the literature on network science.

To further characterize the structural nature of the empirical item-specific networks, we calculate summary statistics for the networks used in the preferential-detachment analyses. For each domain, we report the number of items, the average size of the corresponding item-specific networks, and the estimated power-law exponent of the item-level degree distributions in Table S1. The corresponding degree distributions are shown in Fig. S2. Together, these results show that all four empirical settings exhibit strongly heterogeneous item-level network structures, with

estimated power-law exponents ranging from approximately 1.8 to 3.0.

Table S1: **Structural statistics of the empirical item-specific networks.** For each domain, we construct item-specific relational networks for the focal scientific field, patenting domain, handset model, or drug MOA retained in the main life-cycle analysis. The table reports the number of items, the average size of the corresponding item-level networks, and the estimated power-law exponent γ of the item-level degree distributions.

Domain	Number of items	Average size of item network	Power-law exponent γ
D_1 Science fields	1,219	1,078	2.99
D_2 Patents	291	500	2.98
D_3 Handsets	704	4,926	2.97
D_4 Drug MOAs	248	96	1.79

S2.2 Selecting Time Intervals to Measure the Structural Effect

In Fig. 2 of the main text, we measure the structural effect within a short time interval to control for potential temporal confounding effects. To ensure comparability across the four empirical domains, we focus on the stage of the life cycle when an innovation reaches its half-life, defined as the time at which its popularity has declined to half of its peak level.

For datasets D_1, D_2, and D_4, we measure the structural effect over the minimal time resolution available in the data, around the half-life. For dataset D_3, which records daily user activity, we measure detachment events within a 100-day window around the half-life.

S2.3 Robustness Check Across Different Time Intervals

To examine the robustness of the observed structural effects, we repeat the analyses presented in Fig. 2 of the main text using alternative points along the life-cycle dynamics. In addition to the half-life (50% of peak popularity), we conduct the same analyses when the popularity has declined to 60% (Fig. S3) and 40% (Fig. S4) of its maximum value. The results indicate that the observed patterns remain consistent across these alternative time intervals.

In both Fig. S3 and Fig. S4, we divide individuals and organizations into two groups based on whether detachment events have occurred among their network neighbors ($r_i > 0$ versus $r_i = 0$), where r_i denotes the number of neighbors of node i that have already detached. We then measure

the detachment rate separately for the two groups. The distribution of detachment rates shows visibly different patterns (panels A–D in both figures), with the distribution for the group $r_i > 0$ systematically shifting to the right relative to the $r_i = 0$ group. This pattern indicates that the detachment rate tends to be higher when detachment events have occurred among an individual's network neighbors.

To systematically evaluate this relationship across innovations in different domains, we estimate the relationship between detachment probability ν_i and the number of detached neighbors r_i for each scientific field, patenting domain, mobile handset, and drug MOA. Specifically, we fit a linear model for each innovation and examine the distribution of the estimated slope coefficients. We find that a substantial fraction of innovations across the four domains exhibit a positive slope, indicating that detachment probability increases with the number of neighbors who have already detached. In particular, the fraction of innovations with positive slopes equals 73%, 72%, 79%, and 64% for scientific fields, patenting domains, mobile handsets, and drug MOAs, respectively, when popularity declines to 60% (Fig. S3E–H), and 71%, 57%, 78%, and 60%, respectively, when popularity declines to 40% (Fig. S4E–H).

Together, these results confirm that the structural pattern of preferential detachment remains robust across different points along the life-cycle dynamics.

S2.4 Robustness Check on Averaged Detachment Curves

In addition to the representative item-level examples shown in Fig. 2E–H of the main text, we also calculate averaged detachment curves for each domain. This provides a population-level visualization of the relationship between detachment probability and the number of detached neighbors. As shown in Fig. S5, the averaged curves are consistent with the main empirical pattern reported in Fig. 2.

S2.5 Robustness Check on Normalized Exposure, r_i/k_i

To test whether the observed preferential-detachment pattern is driven only by the raw number of detached neighbors, we repeat the main empirical analysis using normalized exposure, r_i/k_i, where r_i is the number of detached neighbors of node i, and k_i is its degree in the item-specific relational network. This analysis asks whether detachment probability increases with the fraction of a node's neighbors who have already detached. As shown in Fig. S6, the main pattern remains consistent across the four empirical domains, confirming that the observed preferential-detachment pattern is not solely driven by the raw number of detached neighbors.

S2.6 Robustness Check on α Distribution

To connect the empirical detachment patterns to the model parameter α, we estimate an item-level empirical analogue of preferential-detachment strength from the fitted relationship between detachment probability and the number of detached neighbors. Positive fitted slopes correspond to $\alpha > 0$, consistent with preferential rather than random detachment. Figure S7 reports the estimated domain-level values of α, and Table S2 provides the corresponding summary statistics. These results show that the estimated preferential-detachment strength is positive across all four empirical domains.

Table S2: **Summary statistics for empirical preferential-detachment strength.** The table reports, for each empirical domain, the number of items analyzed, the mean slope of the relationship between ν_i and r_i, the percentage of items with positive slopes, and the estimated item-level preferential detachment parameter α. Positive slopes correspond to $\alpha > 0$, consistent with preferential rather than random detachment. Across all four domains, most items exhibit positive slopes, supporting the generality of preferential detachment across scientific, technological, commercial, and pharmacological systems. The table only includes items with a well-defined half-life observation window and within-item variation in exposure. The resulting item counts are therefore a subset of the full item sets reported in Table S1.

Domain	Number of items	Mean slope	Positive slope (%)	Mean item-level α
D_1 Science fields	508	0.0349	73	0.1657
D_2 Patents	125	0.0411	70	0.1410
D_3 Handsets	513	0.0335	79	0.1235
D_4 Drug MOAs	73	0.1390	70	0.3153

S3 Heterogeneous Mean-Field Theory

S3.1 Method Description

To better understand the phase transition phenomenon in the small α region, we adopt the heterogeneous mean-field approximation (HMA)—a frequently used analytical method in epidemiology, network science, and percolation theory[4,5]. HMA assumes that nodes with the same degree follow the same behavior. By denoting i_k as the survival probability of a node with degree k, we can express the fraction of detached nodes f in the system as:

$$f = 1 - \sum_k P_k i_k, \tag{S1}$$

where P_k is the degree distribution.

To identify the critical point f_c at which the giant component vanishes, we calculate u, the probability that a remaining node does not belong to the giant component. u is a function of f satisfying the following equation[5]:

$$u = \sum_k q_k \left(1 - i'_{k+1} + i'_{k+1} u^k\right), \tag{S2}$$

where q_k corresponds to the excess degree distribution of the network, and i'_{k+1} is the *neighbor-weighted survival probability* for nodes with degree $k+1$. This probability quantifies the survival probability of a node with degree $k+1$ when sampled through surviving continuation edges, such that configurations with more surviving continuation neighbors receive proportionally greater weight. Notice that i'_{k+1} is different from i_{k+1}. i_{k+1} is a *direct survival probability*—it

directly counts how many nodes with degree $k+1$ survive, without considering how these nodes are connected to others. i'_{k+1} is more complex. Here, we consider the process of reaching nodes through their neighbors, which introduces a bias: nodes with more connections (i.e., higher-degree nodes) are more likely to be picked multiple times since they have more neighbors. We present the quantitative relationship between i_{k+1} and i'_{k+1} in later sections.

Equation (S2) suggests that if a node is not connected to the giant component, it either links to a node that is also not in the giant component or the node has already been removed. Based on percolation theory, the giant component vanishes when the derivative of the right-hand side equals 1 at $u = 1$. In other words:

$$\left[\frac{d}{du}\sum_k q_k\left(1 - i'_{k+1} + i'_{k+1}u^k\right)\right]_{u=1} = 1. \tag{S3}$$

The phase transition point f_c can be obtained through equation (S3). To achieve this, we need to express i_k in terms of f and substitute it back into equation (S3).

S3.2 Express i_k as a Function of t

Let us first express i_k in terms of t. We have:

$$\frac{di_k}{dt} = -i_k\nu_0[\alpha k\mu(t) + (1-\alpha)]. \tag{S4}$$

Here, ν_0 is an overall time-scale factor in the continuous-time hazard formulation; because the process in the main-text simulations is implemented as a normalized selection probability over

surviving nodes, ν_0 rescales time but does not affect the order of node removals or the critical threshold f_c. $\mu(t) \equiv 1 - \sum_{k'} q_{k'}(i'_{k'+1})$ measures the probability that a remaining node is linked to a neighbor that has detached. Solving equation (S4), we have:

$$i_k = e^{-\nu_0(1-\alpha)t}[h(t)]^k, \tag{S5}$$

where h is a function satisfying:

$$\frac{dh}{dt} = -\nu_0 \alpha \mu h. \tag{S6}$$

By inserting equation (S5) into the definition of μ, we can express μ as a function of h:

$$\mu(t) = 1 - e^{-\nu_0(1-\alpha)t} G_1(h)/h, \tag{S7}$$

where $G_1(x) \equiv \sum_k \frac{1}{<k>}(k+1)P(k+1)x^k$ is the generating function for excess degree. Here we have also used an important relation: $i'_k/i_k = 1/h^2$, which we prove in S3.3. Combining equations (S7) and (S6), we have:

$$\frac{dh}{dt} = -\nu_0 \alpha (h - e^{-\nu_0(1-\alpha)t} G_1(h)). \tag{S8}$$

S3.3 Relationship Between i'_k and i_k

Since i'_k measures the survival probability of k-degree nodes through the nodes' surviving neighbors, nodes with more surviving neighbors will contribute more to the probability. We have:

$$\begin{aligned}
\frac{di'_k}{dt} &= -i'_k \nu_0 \left[(1-\alpha) + \alpha \frac{\sum_{m=0}^{k-1} (k-1-m) m \binom{k-1}{m} (1-\mu)^m \mu^{k-m-1}}{\sum_{m=0}^{k-1} m \binom{k-1}{m} (1-\mu)^m \mu^{k-m-1}} \right] \\
&= -i'_k \nu_0 \left[(1-\alpha) + \alpha \frac{(k-1)^2(1-\mu) - (k-1)(1-\mu)\mu - (k-1)^2(1-\mu)^2}{(k-1)(1-\mu)} \right] \\
&= -i'_k \nu_0 \left[(1-\alpha) + \alpha (k-2)\mu \right].
\end{aligned} \tag{S9}$$

where m measures the number of surviving neighbors of the studied node among the $k-1$ neighbors other than the incoming surviving neighbor. Therefore, $k-1-m$ is the number of removed neighbors. The reason the sum runs from 0 to $k-1$ instead of to k is that the node is reached through a surviving node. Solving this equation, we obtain:

$$i'_k = e^{-\nu_0(1-\alpha)t}[h(t)]^{k-2}. \tag{S10}$$

Combining equations (S10) and (S5), we have:

$$i'_k/i_k = \frac{1}{h^2} \tag{S11}$$

S3.4 Express i_k as a Function of f

Combining equations (S5) and (S1), we have:

$$e^{-\nu_0(1-\alpha)t} = \frac{1-f}{G_0(h)}. \tag{S12}$$

By inserting this result back into equations (S5) and (S8), we have:

$$i_k = \frac{1-f}{G_0(h)}h^k, \tag{S13}$$

and

$$\frac{dh}{df} = \frac{-\alpha h G_0^2(h) + \alpha G_0(h)G_1(h)(1-f)}{\alpha(1-f)hG_0(h)G'_0(h) - \alpha(1-f)^2G'_0(h)G_1(h) + (1-\alpha)(1-f)G_0^2(h)}, \tag{S14}$$

where G_0 and G_1 are generating functions, defined as $G_0(x) \equiv \sum_k P(k)x^k$, $G_1(x) \equiv \sum_k \frac{1}{<k>}(k+1)P(k+1)x^k$. Inserting equation (S13) into equation (S3), we obtain the self-consistent equation

for f_c:

$$f_c = 1 - \frac{G_0(h_c) < k >}{G_0''(h_c)} = 1 - \frac{< k > \sum_k P_k h_c^k}{\sum_k P_k (k^2 - k) h_c^{k-2}}, \tag{S15}$$

where h_c is the solution of equation (S14) for $f = f_c$.

S3.5 Molloy–Reed Criterion Method

An alternative method for identifying the phase transition point is through the Molloy–Reed criterion[3,6]: a network with any degree distribution has a giant component if

$$\frac{< k^2 >}{< k >} > 2. \tag{S16}$$

We first calculate the average degree of a network when a fraction f of the nodes has been removed:

$$\begin{aligned} < k' >_f &= \frac{\sum P_k i_k \binom{k}{k'} k' \mu^{k-k'} (1-\mu)^{k'}}{\sum P_k i_k} \\ &= \frac{\sum_k k h^k P_k (1-\mu)}{G_0(h)} \\ &= \frac{(1-\mu) G_0'(h) h}{G_0(h)} \\ &= \frac{(1-f) G_0'(h) G_1(h)}{G_0^2(h)}. \end{aligned} \tag{S17}$$

In the same way, we can calculate $< k'^2 - k' >$:

$$\begin{aligned} < k'^2 - k' >_f &= \frac{\sum P_k i_k \binom{k}{k'} (k'^2 - k') \mu^{k-k'} (1-\mu)^{k'}}{\sum P_k i_k} \\ &= \frac{\sum_k (k^2 - k) h^k P_k (1-\mu)^2}{G_0(h)} \\ &= (1-f)^2 \frac{G_0''(h) G_1^2(h)}{G_0^3(h)}. \end{aligned} \tag{S18}$$

By combining these results, we have:

$$f_c = 1 - \frac{G_0(h_c) < k >}{G_0'''(h_c)} = 1 - \frac{< k > \sum_k P_k h_c^k}{\sum_k P_k (k^2 - k) h_c^{k-2}}, \tag{S19}$$

which is consistent with our previous findings.

S4 Relationship Between k_f and r

S4.1 Relationship Between k_f and r in Different Network Structures

Why is the relationship between k_f and r different across various networks? An intuitive understanding can be gained by analyzing the initial step of the process, where the first fraction f_0 of nodes is removed from a generated network. The process is a simple Poisson process, where the joint probability of the current degree k and r can be written as: $P_{f_0}(k,r) = P(k+r)\binom{k+r}{r}(1-f)^k f^r$. For an ER network, the formation process is also a Poisson process, where each node is independently connected to other nodes with the same probability. Since each node behaves independently in both the formation and removal processes, the distributions of k and r should also be independent. Mathematically, we have:

$$P_{f_0}(k,r) = \frac{\lambda^{k+r} e^{-\lambda}}{(k+r)!}\binom{k+r}{r}(1-f)^k f^r = \frac{[\lambda(1-f)]^k e^{-\lambda(1-f)}}{k!} \frac{[\lambda f]^r e^{-\lambda f}}{r!} \tag{S20}$$

Here, $P_{f_0}(k,r)$ can be decoupled into two independent distributions for k and r, suggesting that the process can be viewed as two independent processes. In the next section, we show that not

only in the initial step but in all steps of the ER network, the joint probability can be decoupled, demonstrating the independence of the current degree k and r. The key to this decoupling property is the factorial decay of the Poisson process, which must satisfy two criteria: 1) each node is independently attached to other nodes (independence), and 2) each node attaches to other nodes with the same probability (homogeneity).

For a network with heavy tails, the formation process can be represented as an inhomogeneous Poisson process (see hidden variable network models), where each node attaches to others independently but with a different probability. A node with a higher probability is more likely to have a higher r and a higher current degree, presenting a positive correlation between k and r. In contrast, for a network with narrower tails, nodes do not link to others independently, and the degree for each node is bounded. Therefore, removing more neighbors reduces the current degree.

To further understand the process, we directly study the relationship between k_f and r for different f (Fig. 3C in the main text). Interestingly, we find that the curves are parallel to each other when r is relatively large, suggesting that the $k_f - r$ curve is determined only by the network topology in that condition, and it is independent of the specific dynamics. To test this hypothesis, and to systematically uncover the role of network topology in the detachment process, we quantify the relationship between k_f and r for networks with varying levels of degree heterogeneity. We find that although the relationship between k_f and r is complex (see the next section for the full

mathematical derivation), the asymptotic behavior of $k_f(r)$ is very simple:

$$k_f(r)\Big|_{r\to\infty} = W(h)H(r) \sim H(r) \tag{S21}$$

It can be decoupled into two independent factors, $H(r) \equiv \frac{P_k(r+1)}{P_k(r)}(r+1)$, and $P_k(x)$ takes the form of the degree distribution of the network. H quantifies how heavy the degree distribution is compared to a Poisson distribution, and

$$W(h) = \begin{cases} \frac{(1-f)G_1(h)}{G_0(h)-(1-f)G_1(h)} & \text{if } H(r)|_{r\to\infty} \to \infty, \\ (1-f)G_1(h)/G_0(h) & \text{if } H(r)|_{r\to\infty} = H_0. \end{cases} \tag{S22}$$

Since $W(h)$ is a function independent of r, equation (S21) suggests that the trend of $k_f(r)$ is solely determined by H, which depends only on the degree distribution of the network. The second branch includes finite limiting values of H_0, including $H_0 = 0$.

For ER networks, since the degree distribution follows a Poisson distribution (Fig. S8E), we can calculate that $\frac{P_k(x+1)}{P_k(x)} = \frac{\lambda}{x+1}$, and $H(x) = \lambda$, suggesting that $k_f(r)$ remains constant when r is large (Fig. 3F in the main text). Therefore, under preferential detachment, where nodes with higher r are more likely to be detached, we effectively select nodes with a random degree k_f, supporting the conclusion that preferential detachment is identical to random detachment on ER networks. Importantly, for ER networks, $k_f(r)$ remains constant not only for large r but for any given r. This result corroborates our findings in Fig. S8F, explaining why the phase transition point does not change in an ER network. It also echoes the analytical results demonstrated in equation (2) of the main text. The generating functions for ER random networks take special forms: $G_0(x) = G_1(x) = e^{\langle k\rangle(x-1)}$. By substituting this back into equation (2) of the main text,

we recover the Molloy–Reed criterion:

$$f_c = 1 - \frac{\langle k \rangle}{\langle k^2 \rangle - \langle k \rangle},$$

which is independent of α (Fig. 3E in the main text, solid line). This result validates that preferential detachment is exactly the same dynamic process as random detachment on ER networks.

Empirical networks typically exhibit heavy-tailed degree distributions. From equation (4) in the main text, we find that if the degree distribution has a heavier tail than the Poisson distribution, such as a power-law distribution (Fig. S8B), $H(x)$ grows faster than a constant, indicating a positive correlation between k_f and r (Fig. 3C in the main text). Therefore, for these networks, if nodes are removed under the preferential detachment process, hubs are more likely to be removed, leading to an earlier phase transition (Fig. S8C). This result supports the argument that preferential detachment behaves similarly to network attacks (red triangles in Fig. S8C) on heavy-tailed networks. As for networks with narrow degree distributions (i.e., narrower than the Poisson distribution), such as networks with a normal degree distribution (Fig. S8H), $H(x)$ decreases with x, suggesting a negative correlation between k_f and r (Fig. 3I in the main text). Interestingly, the negative relationship between k_f and r indicates that if we remove nodes with higher r under preferential detachment, we are actually targeting nodes with lower remaining degrees, thus inducing a delayed percolation compared to random detachment (Fig. S8I).

Table S3 summarizes the predictions for several common networks:

Table S3: Summary of different network structures

**Network**	**Degree Distribution**	$H(r)$	$H(r)$ **(**$r \to \infty$**)**
Scale-free networks	$A(\gamma)k^{-\gamma}$	$(r+1)(1+1/r)^{-\gamma}$	r
ER networks	$\frac{\lambda^k e^{-\lambda}}{k!}$	λ	λ
Gaussian networks	$A(\mu_0,\sigma)e^{-\frac{1}{2}\left(\frac{k-\mu_0}{\sigma}\right)^2}$	$(r+1)e^{\frac{-2r+2\mu_0-1}{2\sigma^2}}$	0

Note: $A(\gamma)$ and $A(\mu_0,\sigma)$ are normalization constants. Here γ denotes the power-law exponent, λ denotes the mean degree of the Poisson distribution, and μ_0 denotes the mean degree of the Gaussian degree distribution.

S4.2 k_f as a Function of r

In this section, we discuss the average remaining degree of a node after removing a fraction f of nodes, denoted by k_f, as a function of the number of removed neighbors r. We begin our analysis with the random detachment process ($\alpha = 0$) and then proceed to understand the behavior under preferential detachment.

1. Random Detachment ($\alpha = 0$**)**

For random detachment, we have the following expression:

$$\begin{aligned} k_f(r) &= \frac{\sum_{k'=0}^{\infty} k' p(k'+r)\binom{k'+r}{k'} f^r (1-f)^{k'}}{\sum_{k'=0}^{\infty} p(k'+r)\binom{k'+r}{k'} f^r (1-f)^{k'}} \\ &= \frac{G_r^{(r+1)}(1-f)}{G_r^{(r)}(1-f)}(1-f), \end{aligned} \tag{S23}$$

where $G_r(x) = \sum_{k=0}^{\infty} p(k+r)x^{k+r}$, $p(k)$ denotes the degree distribution of the network, and $G_r^{(n)}(x)$ represents the nth derivative of $G_r(x)$, i.e., $G_r^{(n)}(x) = \frac{\partial^n G_r(x)}{\partial x^n}$.

Although $G_r(x)$ is intricate and topology-dependent, its asymptotic behavior can be readily determined. In the limit $r \to \infty$, we have:

$$G_r^{(r)}(x) = \sum_{a=0}^{\infty} (r+a)!\, p(r+a) \frac{x^a}{a!}. \tag{S24}$$

Additionally, we can write:

$$\begin{aligned} G_r^{(r+1)}(x) &= \sum_{a=0}^{\infty} \frac{(r+a)!\, a}{a!} p(r+a) x^{a-1} \\ &= \sum_{a=0}^{\infty} \frac{(r+a+1)!}{a!} p(r+a+1) x^a. \end{aligned} \tag{S25}$$

Now, by combining equations (S24) and (S25), we obtain:

$$\begin{aligned} (1-x)G_r^{(r+1)}(x) &= \sum_{a=0}^{\infty} \left[\frac{(r+a+1)!\, p(r+a+1)}{a!} - \frac{(r+a)!\, a\, p(r+a)}{a!} \right] x^a \\ \Rightarrow \frac{(1-x)G_r^{(r+1)}(x)}{G_r^{(r)}(x)} &= \frac{\sum_{a=0}^{\infty} \left[(r+a)!\, p(r+a) \frac{x^a}{a!} \right] \left[\frac{(r+a+1)!}{(r+a)!} \frac{p(r+a+1)}{p(r+a)} - a \right]}{\sum_{a=0}^{\infty} (r+a)!\, p(r+a) \frac{x^a}{a!}}. \end{aligned} \tag{S26}$$

Let us define $H(r) \equiv (r+1)\frac{p(r+1)}{p(r)}$. Thus, we have:

$$k_f(r) = \frac{1-f}{f} \frac{\sum_{a=0}^{\infty} \left[(r+a)!\, p(r+a) \frac{(1-f)^a}{a!} \right] [H(r+a) - a]}{\sum_{a=0}^{\infty} (r+a)!\, p(r+a) \frac{(1-f)^a}{a!}}. \tag{S27}$$

Next, we consider the asymptotic properties of $k_f(r)$ for networks with varying forms of $H(r)$.

Class 1: $H(r)|_{r\to\infty} \to \infty$

For a network's degree distribution satisfying this condition, $H(r+a)-a \approx H(r)$ as $r \to \infty$. Substituting this into equation (S27), we obtain:

$$k_f(r)|_{r\to\infty} = \frac{1-f}{f} H(r). \tag{S28}$$

Example Class 1: Scale-Free Network

A common example of this class is the scale-free network with a power-law degree distribution:

$$\begin{aligned} p(r) \sim r^{-\gamma} \quad \Rightarrow \quad H(r)|_{r\to\infty} &= (r+1)\left(1+\frac{1}{r}\right)^{-\gamma} \approx (r+1)\left(1-\frac{\gamma}{r}\right) \\ &= r-\gamma+1-\frac{\gamma}{r} = r+O(1) \to \infty. \end{aligned} \tag{S29}$$

Thus, for a scale-free network, we have:

$$k_f(r)|_{r\to\infty} = \frac{1-f}{f} H(r)|_{r\to\infty} = \frac{1-f}{f} r. \tag{S30}$$

Class 2: $H(r)|_{r\to\infty} = H_0$ **(**$H_0 > 0$**)**

For networks with a degree distribution satisfying this condition, we can directly derive:

$$\begin{aligned}
k_f(r)|_{r\to\infty} &= \frac{1-f}{f}\frac{\sum_{a=0}^{\infty}\left[(r+a)!\,p(r+a)\frac{(1-f)^a}{a!}\right][H(r+a)-a]}{\sum_{a=0}^{\infty}(r+a)!\,p(r+a)\frac{(1-f)^a}{a!}} \\
&= \frac{1-f}{f}(H_0 - \frac{\sum_{a=0}^{\infty}(r+a)!\,p(r+a)\frac{(1-f)^a}{a!}(a)}{\sum_{a=0}^{\infty}(r+a)!\,p(r+a)\frac{(1-f)^a}{a!}}) \\
&= \frac{1-f}{f}(H_0 - \frac{\sum_{a=0}^{\infty}H_0^{r+a}\frac{(1-f)^a}{a!}(a)}{\sum_{a=0}^{\infty}H_0^{r+a}\frac{(1-f)^a}{a!}}) \\
&= \frac{1-f}{f}(H_0 - \frac{\sum_{a=0}^{\infty}H_0^{r+a}\frac{(1-f)^a}{a!}a}{\sum_{a=0}^{\infty}H_0^{r+a}\frac{(1-f)^a}{a!}}) \\
&= \frac{1-f}{f}(H_0 - \frac{\sum_{a=0}^{\infty}H_0^{a}\frac{(1-f)^a}{a!}a}{\sum_{a=0}^{\infty}H_0^{a}\frac{(1-f)^a}{a!}}) \\
&= \frac{1-f}{f}(H_0 - \frac{e^{H_0(1-f)}H_0(1-f)}{e^{H_0(1-f)}}) \\
&= (1-f)H_0 = (1-f)H(r)
\end{aligned} \tag{S31}$$

Example Class 2: Erdős–Rényi (ER) Network

For the Erdős–Rényi (ER) network, where the degree distribution follows a Poisson distribution with mean degree λ, $H(r)$ is constant:

$$p(r) \sim \frac{e^{-\lambda}\lambda^r}{r!} \quad \Rightarrow \quad H(r) = (r+1)\frac{\lambda}{r+1} = H_0 = \lambda. \tag{S32}$$

Since $H(r)$ remains constant for all r, we can calculate $G_r(x)$ for any r:

$$\begin{aligned}
G_r(x) &= \sum_{k=0}^{\infty} p(k+r)x^{k+r} \\
&\sim \sum_{k=0}^{\infty}\frac{e^{-\lambda}\lambda^{k+r}}{(k+r)!}x^{k+r} \\
&= e^{-\lambda}\left(e^{\lambda x} - \sum_{n=0}^{r-1}\frac{(\lambda x)^n}{n!}\right).
\end{aligned} \tag{S33}$$

Taking the rth and $(r+1)^{\text{th}}$ derivatives of the second term results in its vanishing. Therefore, we have:

$$k_f(r) = \lambda(1-f) = (1-f)H_0, \tag{S34}$$

which is consistent with equation (S31).

Class 3: $H(r)|_{r\to\infty} = 0$

For networks satisfying this condition, we revisit the definitions of $G_r^{(r)}$ and $G_r^{(r+1)}$. For

$$G_r^{(r)}(x) = \sum_{a=0}^{\infty}(r+a)!\,p(r+a)\frac{x^a}{a!}, \tag{S35}$$

we analyze the ratio of consecutive terms, yielding:

$$\frac{(r+a+1)!\,p(r+a+1)\frac{x^{a+1}}{(a+1)!}}{(r+a)!\,p(r+a)\frac{x^a}{a!}}|_{r\to\infty} = \frac{x}{1+a}H(r)|_{r\to\infty} = 0. \tag{S36}$$

Thus, only the first term dominates. The same applies to $G_r^{(r+1)}$, leading to:

$$G_r^{(r)}(x)|_{r\to\infty} = r!p(r), \tag{S37}$$

and

$$G_r^{(r+1)}(x)|_{r\to\infty} = (r+1)!p(r+1). \tag{S38}$$

By substituting these expressions into the definition of $k_f(r)$, we find:

$$k_f(r)|_{r\to\infty} = (1-f)(r+1)\frac{p(r+1)}{p(r)} = (1-f)H(r). \tag{S39}$$

Example Class 3: Normal Degree Distribution

For a network with a normal degree distribution:

$$p(k) \sim e^{-\frac{1}{2}\left(\frac{k-\mu_0}{\sigma}\right)^2}, \tag{S40}$$

we find:

$$H(r) = (r+1) \cdot \exp\left(\frac{-2r + 2\mu_0 - 1}{2\sigma^2}\right),$$

and therefore,

$$k_f(r)|_{r\to\infty} = (1-f)H(r)|_{r\to\infty} = 0. \tag{S41}$$

Summary for Random Detachment

In summary, for random detachment, the asymptotic behavior of $k_f(r)$ across the different classes can be expressed as:

$$k_f(r)\Big|_{r\to\infty} = L(f)H(r) \sim H(r), \tag{S42}$$

where $H(r) \equiv \frac{P_k(r+1)}{P_k(r)}(r+1)$ and

$$L(f) = \begin{cases} (1-f)/f & \text{if } H(r)|_{r\to\infty} \to \infty, \\ (1-f) & \text{if } H(r)|_{r\to\infty} = H_0. \end{cases} \tag{S43}$$

2. Preferential Detachment ($\alpha > 0$)

Next, we consider k_f as a function of r under preferential detachment. We have

$$k_f(r) = \frac{\sum_{k'=0}^{\infty} p(k'+r) i_{k'+r} \binom{k'+r}{k'} k' \mu^r (1-\mu)^{k'}}{\sum_{k'=0}^{\infty} p(k'+r) i_{k'+r} \binom{k'+r}{k'} \mu^r (1-\mu)^{k'}} \tag{S44}$$

where $i_k(f) \sim h^k$ is a function of f representing the probability that a node of degree k remains when a fraction f of nodes has been removed. Here, μ is the probability that a neighbor of a node has been removed.

$$\begin{aligned} k_f(r) &= \frac{\sum_{k'=0}^{\infty} p(k'+r)\binom{k'+r}{k'} k'[(1-\mu)h]^{k'}}{\sum_{k'=0}^{\infty} p(k'+r)\binom{k'+r}{k'}[(1-\mu)h]^{k'}} \\ &= \frac{G_r^{(r+1)}\big(h(1-\mu)\big)}{G_r^{(r)}\big(h(1-\mu)\big)} h(1-\mu) \end{aligned} \tag{S45}$$

Combining equations (S7) and (S12), we have:

$$\mu = 1 - \frac{G_1(h)}{h}\frac{1-f}{G_0(h)}. \tag{S46}$$

Since only functions of f are replaced in this equation, the asymptotic behavior of k_f depends solely on the network topology.

Therefore, we obtain:

$$k_f(r)\Big|_{r\to\infty} = W(h)H(r) \sim H(r), \tag{S47}$$

where

$$W(h) = \begin{cases} \frac{(1-f)G_1(h)}{G_0(h)-(1-f)G_1(h)} & \text{if } H(r)|_{r\to\infty} \to \infty, \\ (1-f)G_1(h)/G_0(h) & \text{if } H(r)|_{r\to\infty} = H_0, \end{cases} \tag{S48}$$

and h is a function of f.

We have again:

$$k_f(r)\Big|_{r\to\infty} \sim H(r) = (r+1)\frac{p(r+1)}{p(r)}. \tag{S49}$$

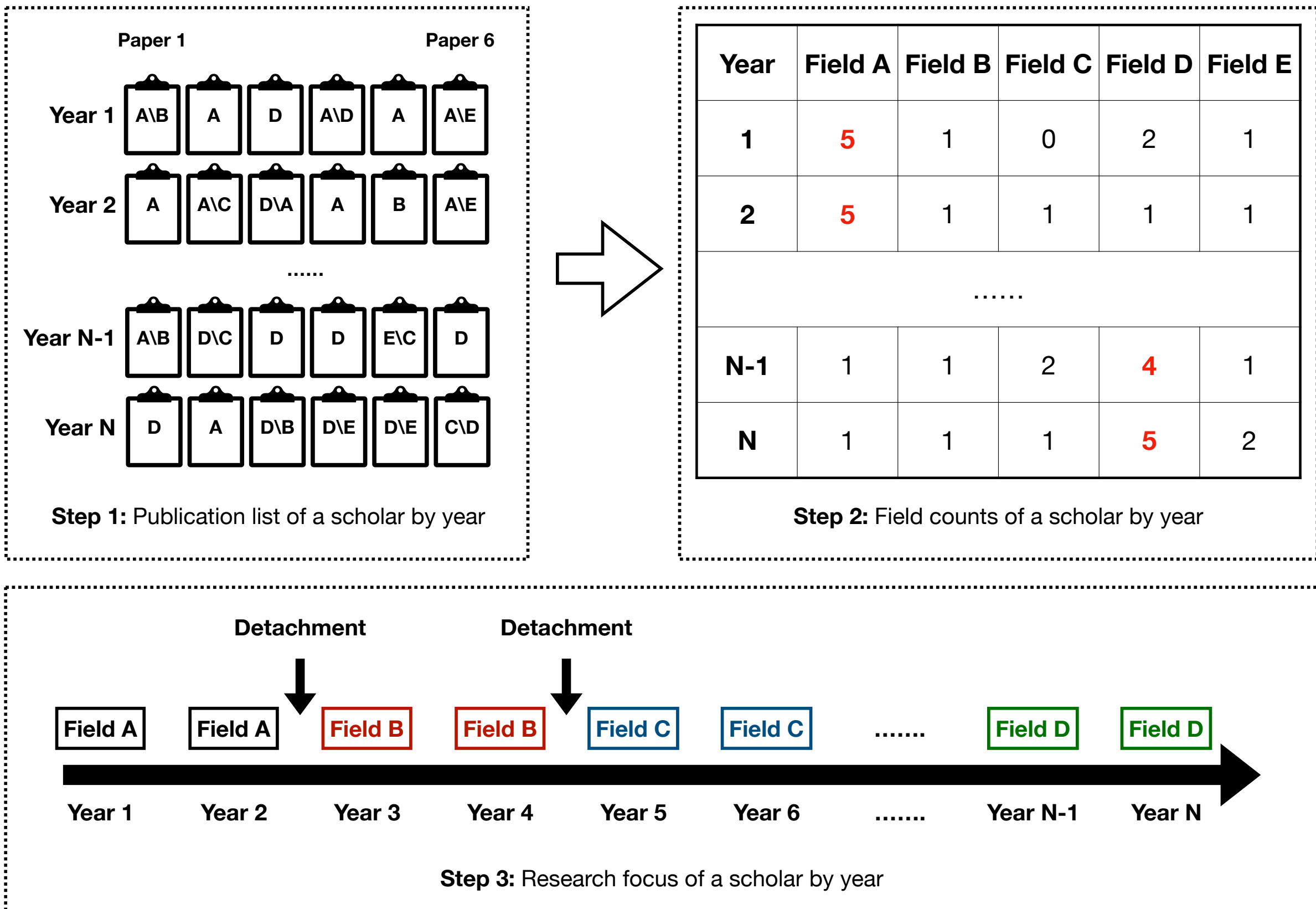

Figure S1: **Example of a scientist's field detachments**.

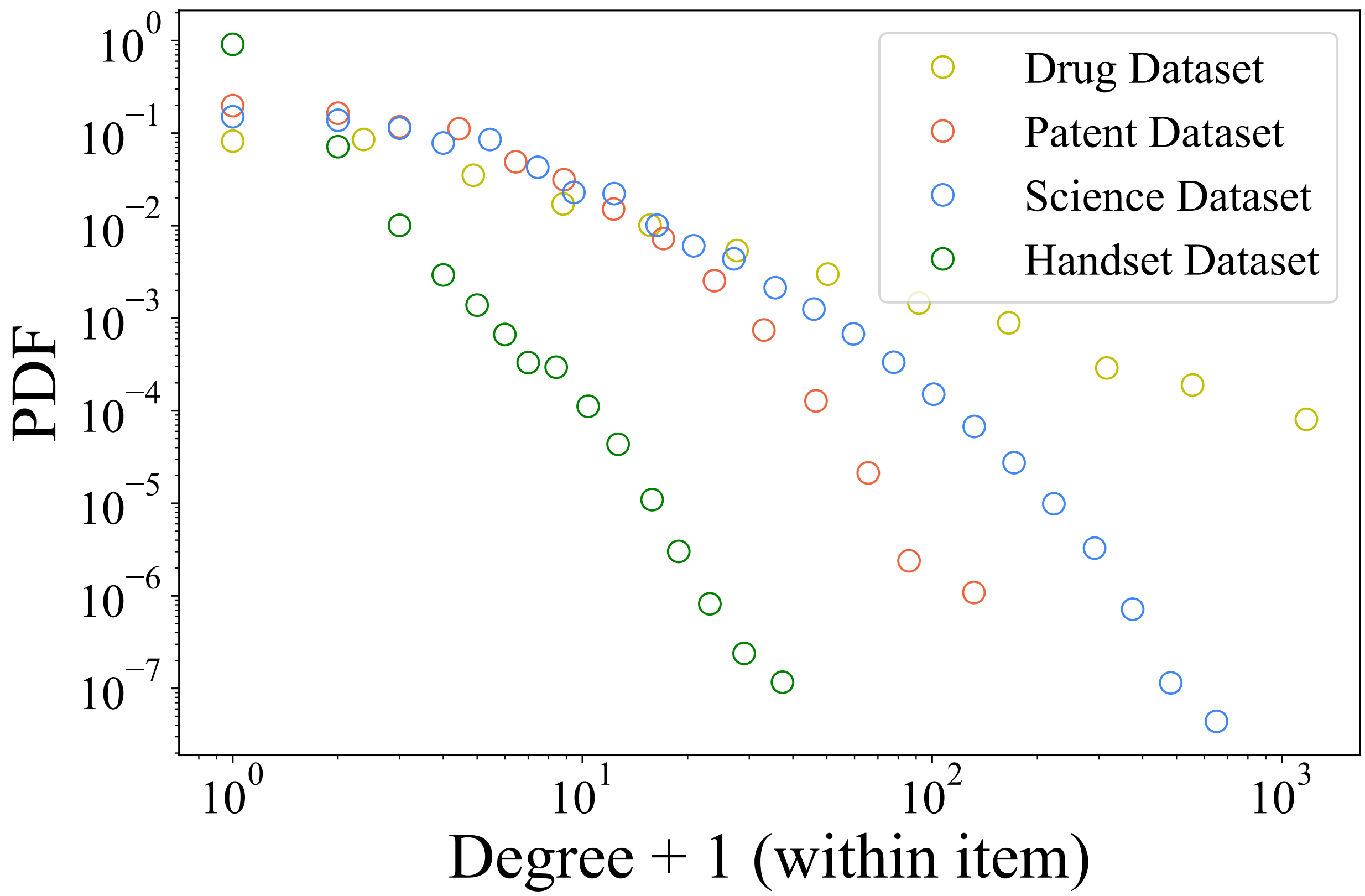

Figure S2: **Degree distributions of the empirical item-specific networks.** The figure plots the degree distributions of item-specific relational networks across the four domains: scientific fields, patenting domains, mobile handsets, and drug MOAs. In each domain, networks are constructed among actors associated with the same focal item using the corresponding underlying relation, including collaboration, communication, or market-focus similarity. The degree distributions are heavy-tailed across all four settings, indicating substantial heterogeneity in local connectivity and motivating the explicit treatment of network topology in the analysis of detachment dynamics.

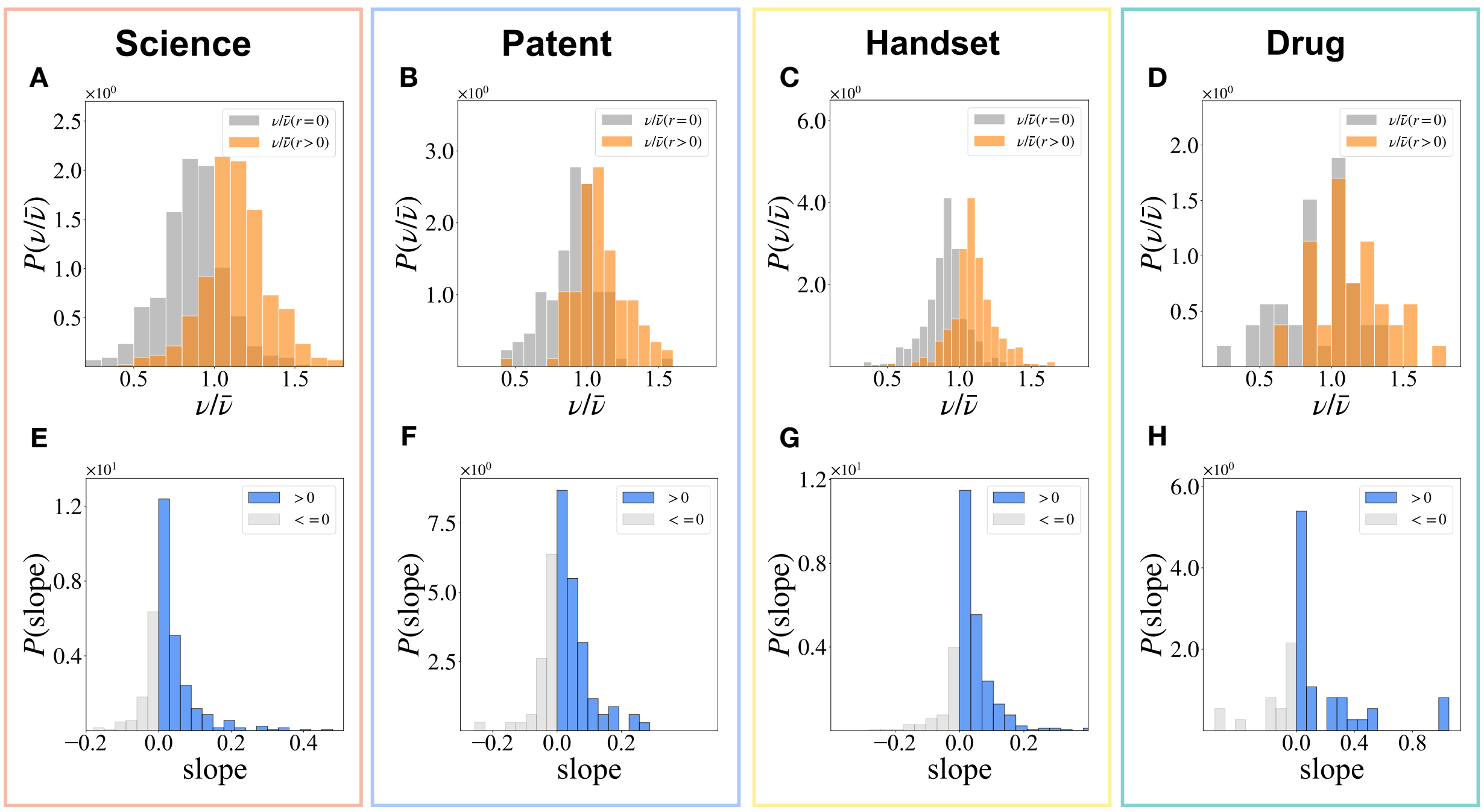

Figure S3: **Robustness check across different time intervals (reduced to 60% of maximum)**.

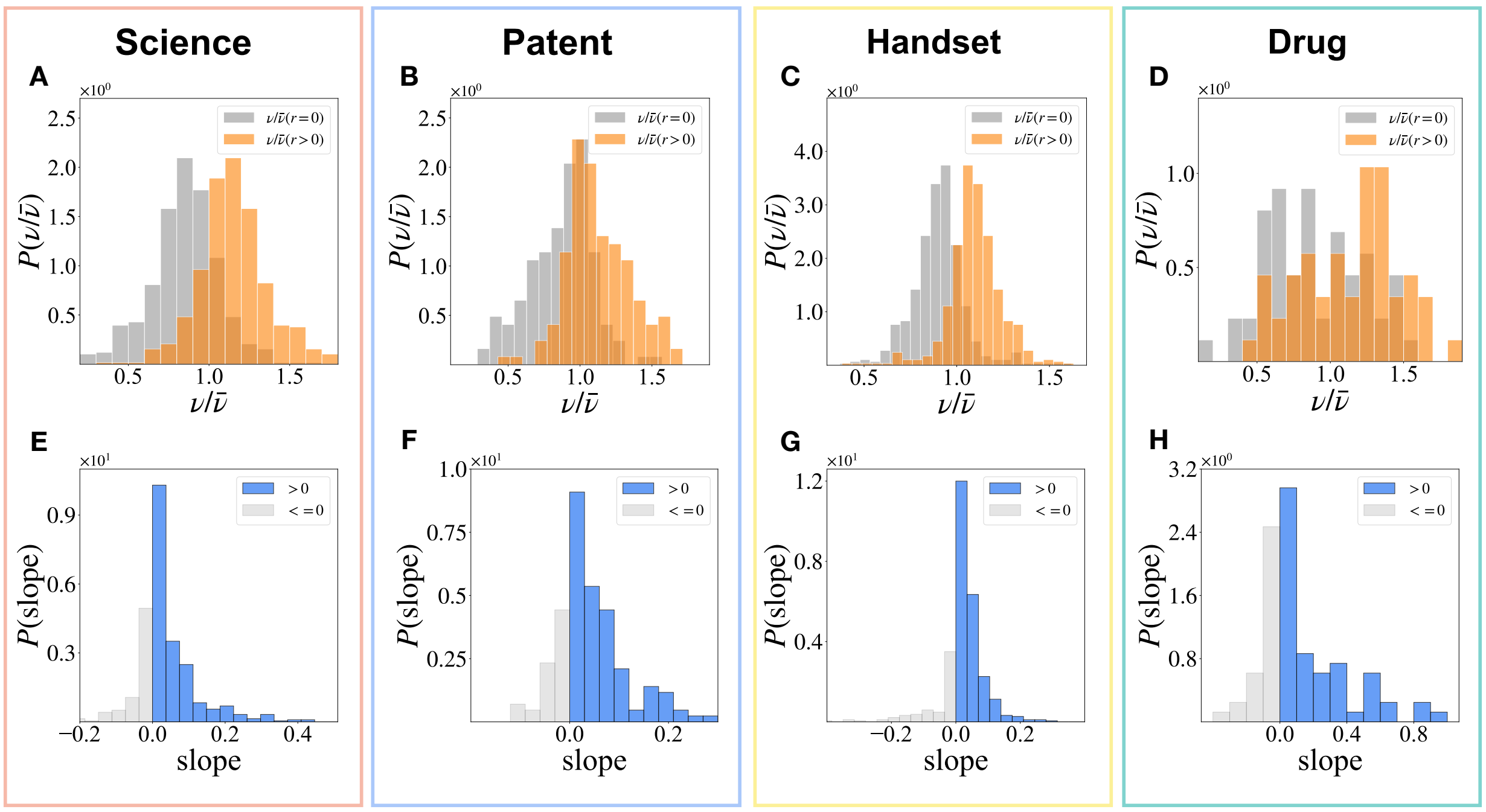

Figure S4: **Robustness check across different time intervals (reduced to 40% of maximum)**.

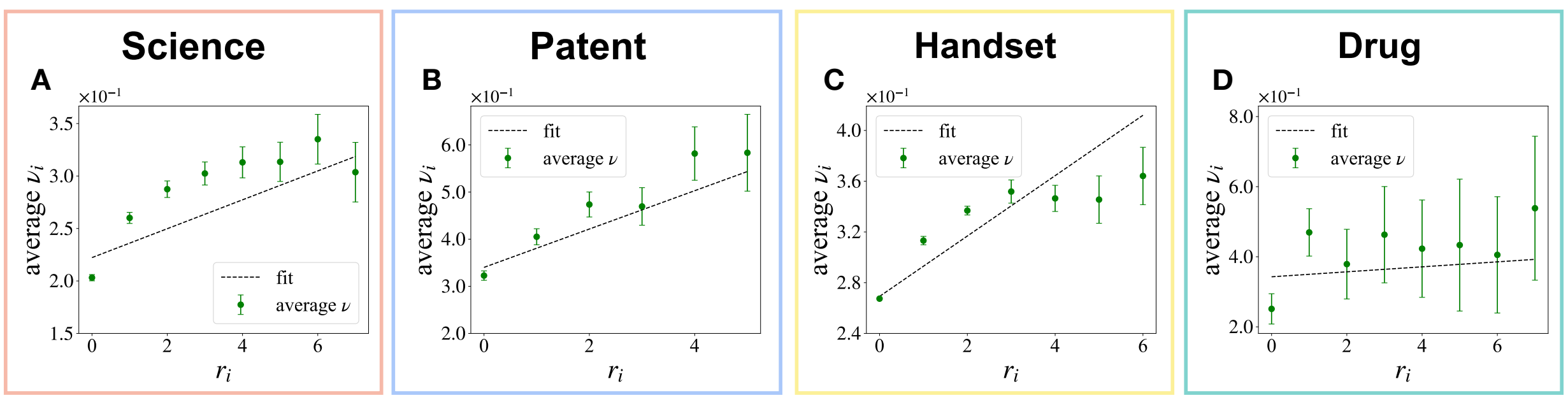

Figure S5: **Averaged detachment curves across the four empirical domains.** In addition to the representative item-level examples shown in Fig. 2E–H of the main text, we calculate averaged curves showing the relationship between detachment probability and the number of detached neighbors for each domain. The averaged curves provide a population-level visualization and show patterns consistent with the main empirical results in Fig. 2.

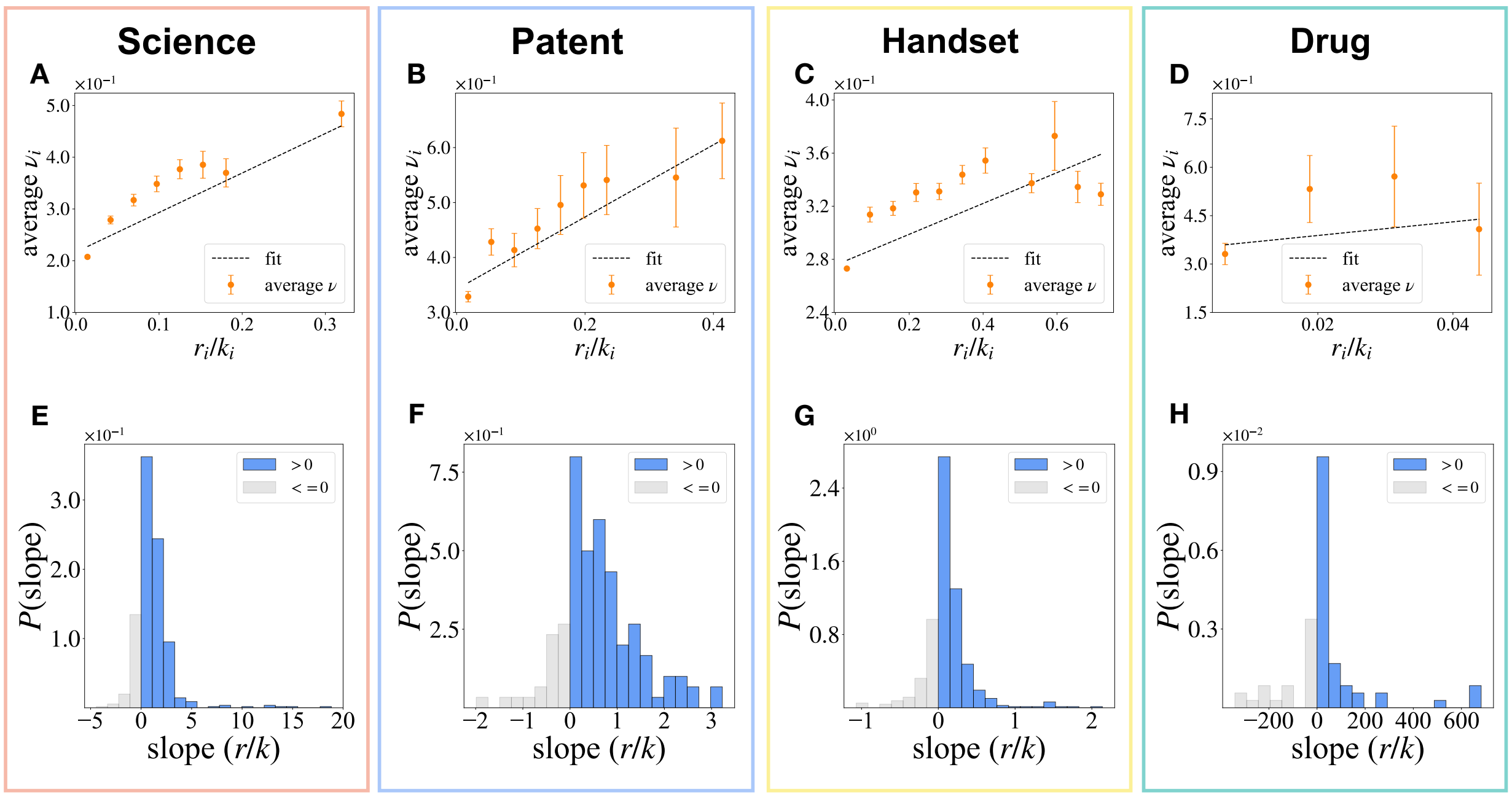

Figure S6: **Robustness of preferential detachment using normalized exposure.** We repeat the main empirical analysis using normalized exposure, r_i/k_i, defined as the fraction of node i's neighbors who have already detached from the focal item. Across the four empirical domains, the detachment probability ν_i increases with normalized exposure, confirming that the observed preferential-detachment pattern is not solely driven by the raw number of detached neighbors.

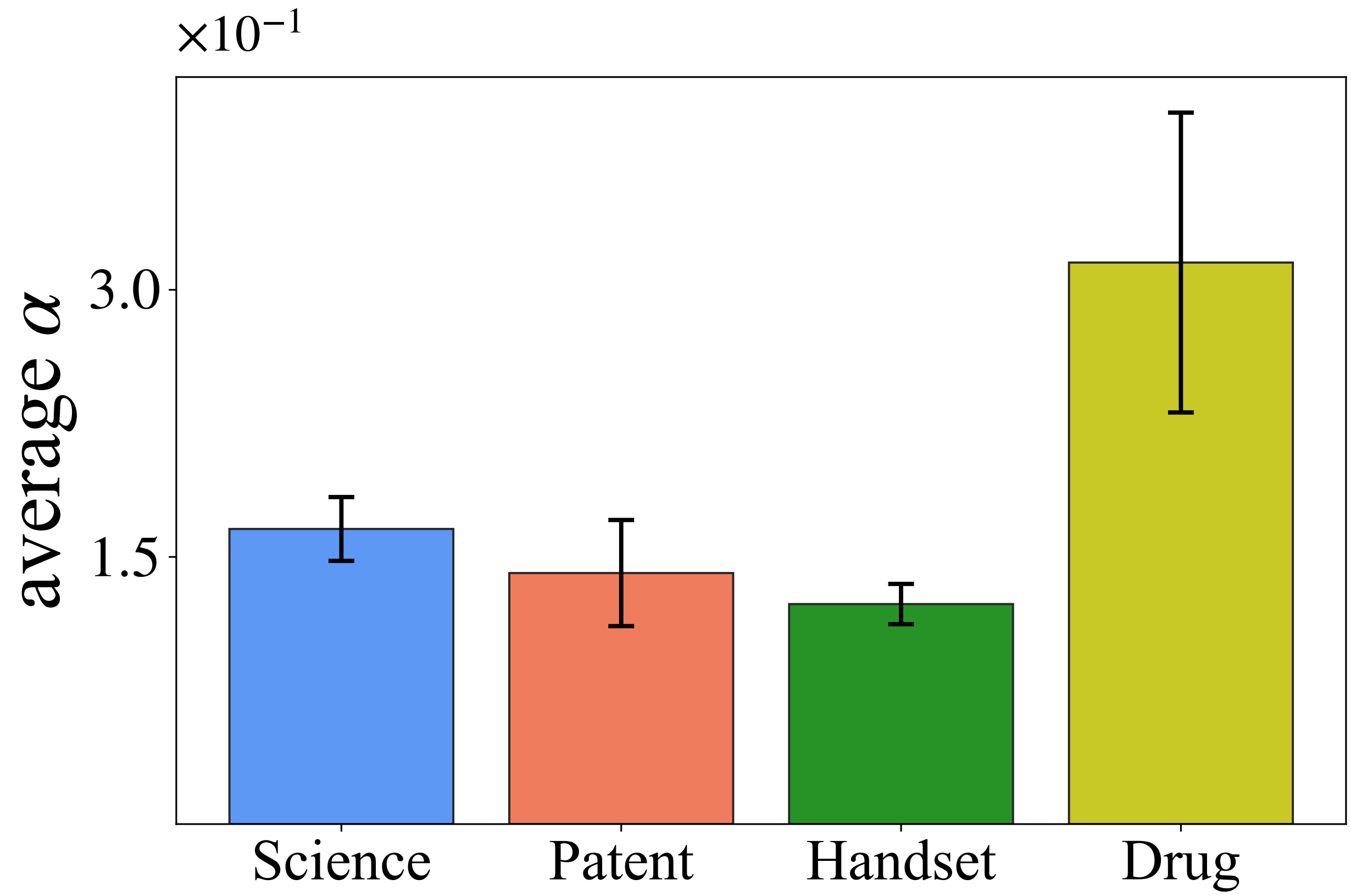

Figure S7: **Empirical estimates of preferential-detachment strength across domains.** We estimate an item-level empirical analogue of α from the fitted relationship between detachment probability and the number of detached neighbors. The figure reports the average estimated α values across the four empirical domains, with error bars indicating 95% confidence intervals.

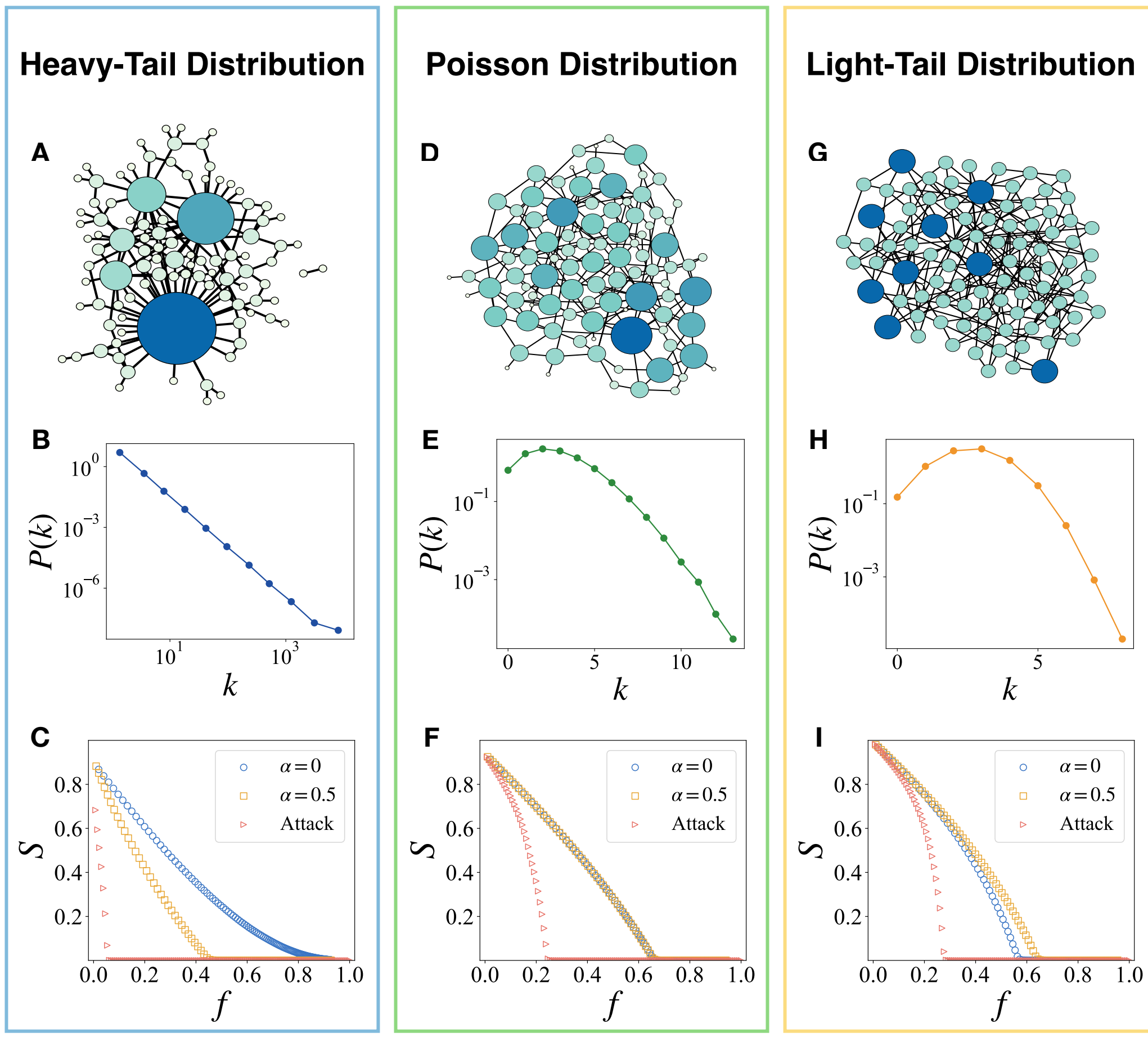

Figure S8: **Percolation process in different network topologies.**

S5 Supplementary References

1. Sinha, A. *et al.* An overview of Microsoft Academic Service (MAS) and applications. In *Proceedings of the 24th International Conference on World Wide Web*, 243–246 (ACM, 2015).

2. Wang, K. *et al.* Microsoft Academic Graph: When experts are not enough. *Quantitative Science Studies* **1**, 396–413 (2020).

3. Barabási, A.-L. *Network Science* (Cambridge University Press, 2016).

4. Pastor-Satorras, R., Castellano, C., Van Mieghem, P. & Vespignani, A. Epidemic processes in complex networks. *Reviews of Modern Physics* **87**, 925 (2015).

5. Newman, M. *Networks* (Oxford University Press, 2018).

6. Molloy, M. & Reed, B. A critical point for random graphs with a given degree sequence. *Random Structures & Algorithms* **6**, 161–180 (1995).
